# *Mycobacterium tuberculosis* infection modulates adipose tissue biology

**DOI:** 10.1371/journal.ppat.1006676

**Published:** 2017-10-17

**Authors:** Macarena Beigier-Bompadre, Georgina N. Montagna, Anja A. Kühl, Laura Lozza, January Weiner, Andreas Kupz, Alexis Vogelzang, Hans-Joachim Mollenkopf, Delia Löwe, Silke Bandermann, Anca Dorhoi, Volker Brinkmann, Kai Matuschewski, Stefan H. E. Kaufmann

**Affiliations:** 1 Department of Immunology, Max Planck Institute for Infection Biology, Berlin, Germany; 2 Parasitology Unit, Max Planck Institute for Infection Biology, Berlin, Germany; 3 Medical Department, Division of Gastroenterology, Infectiology and Rheumatology, Charité - University Medicine, Berlin, Germany; 4 Core Facility, Max Planck Institute for Infection Biology, Berlin, Germany; McGill UniversityHealth Centre, CANADA

## Abstract

*Mycobacterium tuberculosis* (Mtb) primarily resides in the lung but can also persist in extrapulmonary sites. Macrophages are considered the prime cellular habitat in all tissues. Here we demonstrate that Mtb resides inside adipocytes of fat tissue where it expresses stress-related genes. Moreover, perigonadal fat of Mtb-infected mice disseminated the infection when transferred to uninfected animals. Adipose tissue harbors leukocytes in addition to adipocytes and other cell types and we observed that Mtb infection induces changes in adipose tissue biology depending on stage of infection. Mice infected via aerosol showed infiltration of inducible nitric oxide synthase (iNOS) or arginase 1 (Arg1)-negative F4/80^+^ cells, despite recruitment of CD3^+^, CD4^+^ and CD8^+^ T cells. Gene expression analysis of adipose tissue of aerosol Mtb-infected mice provided evidence for upregulated expression of genes associated with T cells and NK cells at 28 days post-infection. Strikingly, IFN-γ-producing NK cells and Mtb-specific CD8^+^ T cells were identified in perigonadal fat, specifically CD8^+^CD44^-^CD69^+^ and CD8^+^CD44^-^CD103^+^ subpopulations. Gene expression analysis of these cells revealed that they expressed IFN-γ and the lectin-like receptor *Klrg1* and down-regulated *CD27* and *CD62L*, consistent with an effector phenotype of Mtb-specific CD8^+^ T cells. Sorted NK cells expressed higher abundance of *Klrg1* upon infection, as well. Our results reveal the ability of Mtb to persist in adipose tissue in a stressed state, and that NK cells and Mtb-specific CD8^+^ T cells infiltrate infected adipose tissue where they produce IFN-γ and assume an effector phenotype. We conclude that adipose tissue is a potential niche for Mtb and that due to infection CD8^+^ T cells and NK cells are attracted to this tissue.

## Author summary

In 2015, tuberculosis (TB) affected 10.4 million individuals causing 1.8 million deaths per year. Yet, a much larger group– 2 billion people–harbors latent TB infection (LTBI) without clinical symptoms, but at lifelong risk of reactivation. The physiological niches of *Mycobacterium tuberculosis* (Mtb) persistence remain incompletely defined and both pulmonary and extrapulmonary sites have been proposed. Adipose tissue constitutes 15–25% of total body mass and is an active production site for hormones and inflammatory mediators. The increasing prevalence of obesity, has led to greater incidence of type 2 diabetes. These patients suffer from three times higher risk of developing TB, pointing to a potential link between adipose tissue and TB pathogenesis. In individuals with LTBI, Mtb survives in a stressed, non-replicating state with low metabolic activity and resting macrophages serve as preferred habitat and become effectors after appropriate stimulation. Here we demonstrate that Mtb can infect and persist within adipocytes where it upregulates stress-related genes. In vivo, relative proportions of leukocyte subsets infiltrating adipose tissue varied under different conditions of infection. During natural aerosol Mtb infection, distinct leukocyte subsets, including mononuclear phagocytes, Mtb-specific CD8^+^ T cells and NK cells infiltrated adipose tissue and became activated. Thus, our study shows that adipose tissue is not only a potential reservoir for this pathogen but also undergoes significant alteration during TB infection.

## Introduction

In 2015, tuberculosis (TB) affected 10.4 million individuals leading to 1.8 million deaths globally [1, 2]. TB is primarily a disease of the lung, which serves as port of entry and site of disease manifestation. In the lung, *Mycobacterium tuberculosis* (Mtb) is preferentially entrapped in granulomas [3]. Approximately one-third of the global population suffers from latent TB infection, which persists without apparent clinical signs of disease, and which can be reactivated to active TB at later time points [4]. The niches where Mtb persists remain incompletely understood and both pulmonary and extrapulmonary sites have been proposed [5]. Adipose tissue, which harbors pathogens such as *Trypanosoma cruzi* [6, 7] constitutes 15–25% of the total body mass [8] and is a rich source of hormones and inflammatory cytokines that participate in host defence against infectious agents [6, 9]. In vitro studies revealed that murine adipocytes release TNF, IL-6, IL-12p40 and IL-10 upon Mtb infection [10]. These cells are also susceptible to infection with *Chlamydia pneumoniae*, influenza A, respiratory syncytial virus [11] and human immunodeficiency virus (HIV) [12, 13]. While some epidemiological studies suggest that obesity is inversely associated with TB [14–16], others have found a positive genetic association between the two diseases [17]. Obesity and changing patterns of diet are associated with type 2 diabetes, and complex interrelations between nutrition, obesity, diabetes, and TB are increasingly appreciated [18]. Therefore, we embarked on a systematic in vivo study towards better understanding of the role of adipose tissue in Mtb infection.

Resting macrophages are the preferred habitat of Mtb, which turn into effector cells after appropriate activation. Adipose tissue includes diverse cell types such as monocytes, F4/80^+^ macrophages [19], CD4^+^ and CD8^+^ T cells [20, 21], endothelial cells, and vascular smooth muscle cells [6] and proportions of these cell populations vary under different pathophysiologic conditions [19, 20]. Here, we identified Mtb in adipocytes of fat tissue after aerosol infection of mice and expression of stress-related genes in Mtb within human and mouse adipocytes. We also demonstrated the capacity of adipose tissue to carry Mtb when transferred to uninfected animals. Finally, we identified Mtb-specific effector CD8^+^ T cells and NK cells expressing IFN-γ in adipose tissue after aerogenic Mtb infection. We conclude that adipose tissue provides a potential sanctuary for Mtb in vivo and that Mtb persistence markedly affects adipose tissue biology.

## Results

### Mtb expresses stress-related genes in adipocytes

**Fig 1:**
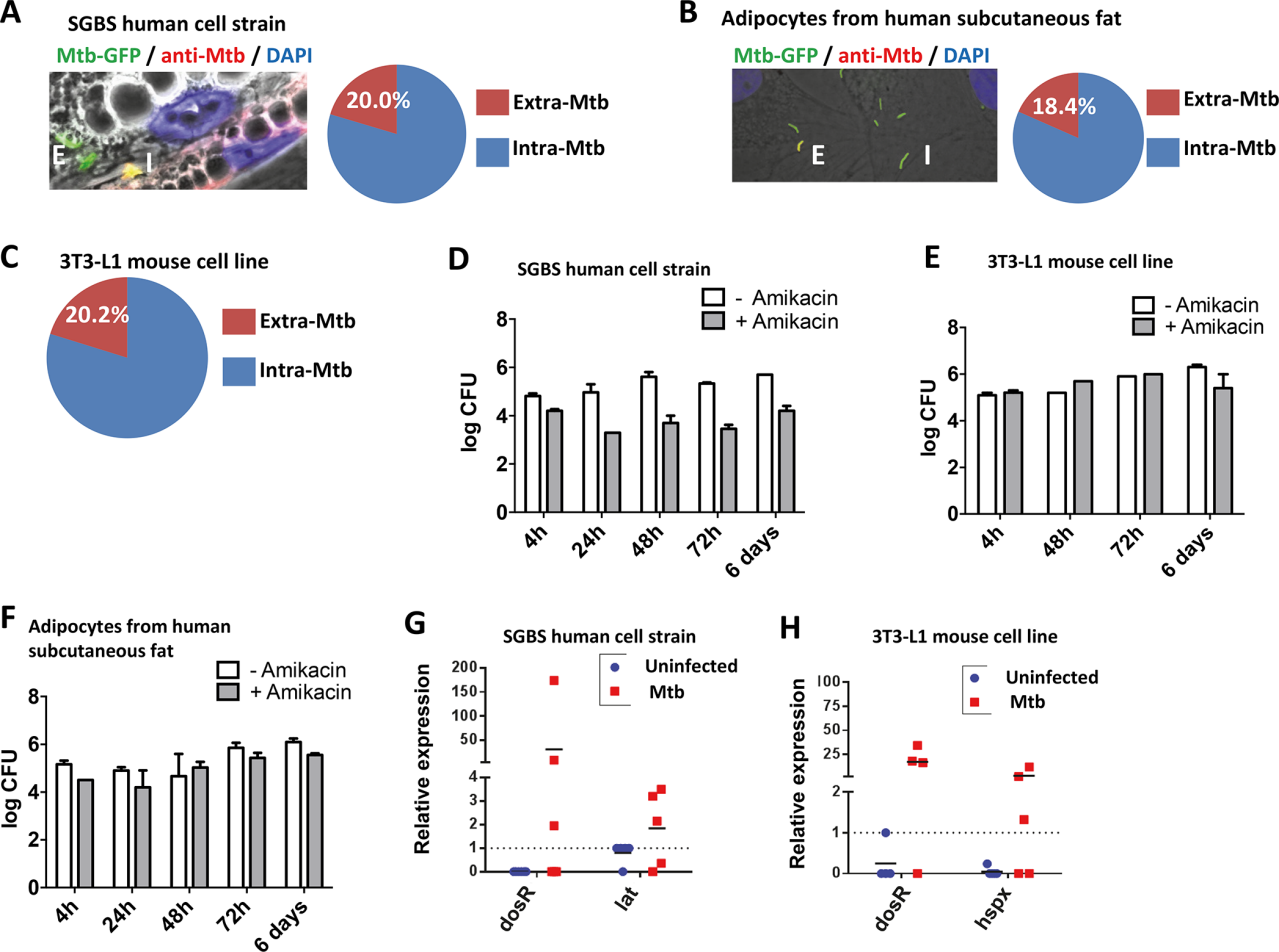
Mtb infects human and mouse adipocytes in vitro and expresses stress-related genes. (**A-C**) Immunofluorescence staining and quantification of extra- and intracellular Mtb-GFP at multiplicity of infection (MOI) of 20 after 24 h in (**A**) SGBS human cell strain; (**B**) primary adipocytes from human subcutaneous fat; (**C**) 3T3-L1 mouse cell line. Blue: DAPI. Original magnification 1,000×. Data are representative of two independent experiments (triplicates). (**D-F**) log_10_ Mtb CFUs at indicated time points post-infection with MOI = 5 in the presence or absence of amikacin in (**D**) SGBS human cell strain, (**E**) 3T3-L1 mouse cell line, (**F**) primary adipocytes from human subcutaneous fat; data are representative of two to four independent experiments. (**G-H**) Expression of Mtb genes *dosR*, *hspX* and *lat* in (**G**) SGBS human cell strain and (**H**) 3T3-L1 mouse cell line. Cells were infected with MOI = 5. Steady state transcript levels were normalized to Mtb *sigA* expression. Means of duplicates from four to six independent experiments are shown. Abbreviations: E, extracellular; I, intracelular; SGBS, Simpson-Golabi-Behmel syndrome.

When human and murine adipocytes were cultured with Mtb in vitro, approximately 80% of the bacterial inoculum was engulfed within 24 h (Fig 1A, 1B and 1C), consistent with published data on Mtb uptake by mouse adipocytes [5]. In a control experiment, professional phagocytes, such as the human macrophage cell line THP-1, took up nearly 98% bacteria under comparable conditions. To determine whether Mtb can replicate inside human and murine adipocytes we counted CFUs between 4 h and 6 days post-infection in presence or absence of the cell-impermeable antibiotic amikacin. Over time, numbers of CFUs remained constant indicating that Mtb did not replicate inside adipocytes (Fig 1D, 1E and 1F), again corroborating previous findings [5]. During persistence in the host under stress conditions [22], Mtb becomes dormant and expresses a specific gene program under the control of the dormancy survival regulon (*dosR*, *Rv3133c*) [23, 24]. These genes include *hspX* (*Rv2031*), which encodes alpha-crystallin, and *lat* (*Rv3290c*) encoding a lysine aminotransferase [25], which are considered as mycobacterial stress markers [26]. In Mtb-infected human adipocytes after 48 h, both *dosR* and *lat* were upregulated (Fig 1G) while in the murine cell line 3T3-L1, *dosR* and *hspX* were induced (Fig 1H). We note that relative expression levels varied between experiments, likely reflecting biological variations in commitment to dormancy and conclude that Mtb is internalized by human and mouse adipocytes where it ceases from replication and becomes stressed.

### After aerosol infection of mice Mtb resides in perigonadal fat

**Fig 2:**
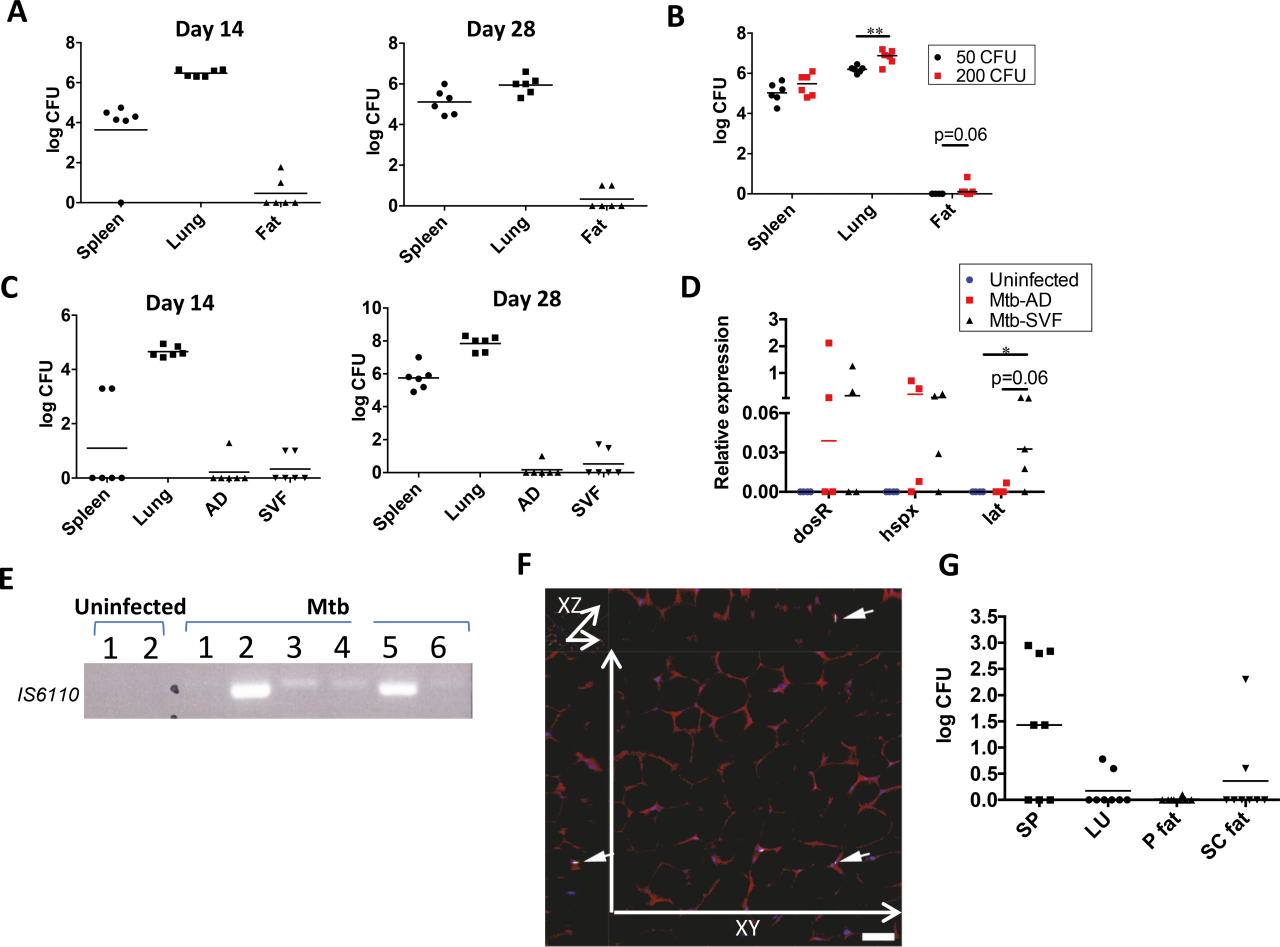
Mtb expresses stress-related genes in perigonadal fat at different time points post aerosol-infection of mice. (**A**) log_10_ Mtb CFUs in spleen, lung and perigonadal fat at different time points post aerosol-infection (200 CFUs). Data are representative of four independent experiments (medians). (**B**) log_10_ Mtb CFUs in spleen, lung and perigonadal fat at day 28 post aerosol-infection with 50 or 200 CFUs. Data are representative of two independent experiments (median); ** p<0.01 (Mann–Whitney test). (**C**) log_10_ Mtb CFUs in spleen, lung and AD and SVF fractions of perigonadal fat at different time points after aerosol infection. Data representative of two independent experiments (medians). (**D**) Expression of Mtb genes *dosR*, *hspX* and *lat* in the AD and SVF fractions of perigonadal fat at day 28 post aerosol-infection. Results of four independent experiments pooled (medians); *p<0.05 (Mann–Whitney test). (**E**) PCR specific for Mtb (IS 6110) in perigonadal fat at day 14 post aerosol-infection. Each lane represents a different mouse. Data representative of two independent experiments. (**F**) Immunofluorescence staining of perigonadal fat of mice infected i.v. with Mtb-GFP at 48 h after infection (arrows, green). Blue: DNA-intercalating dye Draq5. Data representative of two independent experiments. (**G**) log_10_ Mtb CFUs in spleen, lung, perigonadal (P) and subcutaneous (SC) fat from uninfected mice that received a subcutaneous injection of pergonadal fat from mice previously infected with 5x10^6^ CFUs of Mtb. Organs were collected 14 days after the transfer of perigonadal fat. Data representative of two independent experiments (medians). Abbreviations: AD, adipose fraction; P, perigonadal; SC, subcutaneous; SVF, stromal vascular fraction.

We next evaluated whether Mtb resides in adipose tissue after aerosol infection of mice. For these experiments we evaluated the Mtb-CFUs present in the whole perigonadal fat pad, which was extensively washed to exclude possible blood contamination. Perigonadal fat pad was evaluated because obesity studies have reported its association with insulin resistance [27, 28]. At 14 and 28 days post-infection, CFUs of Mtb were not only detected in lung and spleen but also in perigonadal fat of infected mice (Fig 2A). These organs also harbored Mtb at 56 and 90 days post-infection (S1A Fig). Mtb was detectable in perigonadal fat of 17% to 83% of mice at 14 and 28 days post-infection throughout the experiments, while dissemination from lung to spleen was observed in all animals at these time points. The abundance of Mtb in perigonadal fat tended to increase with inoculum dose via aerosol (50 CFUs: no colonies; 200 CFUs: logCFUs from 0.1 to 0.9; 4 out of 6 animals showed colonies) (Fig 2B). Mtb-CFUs were also observed in subcoutaneous fat after aerosol infection (S1B Fig). In addition to adipocytes, fat tissue contains numerous cell types including macrophages [19] and T cells [20, 21], which together form the stromal vascular fraction (SVF). When adipose fraction and SVF were mechanically and enzymatically separated, Mtb was identified both in the adipose fraction and in the SVF between 14 and 28 days post-infection (Fig 2C) indicating that adipose cells as well as leukocytes present in the fat tissue harbor Mtb. To investigate whether Mtb residing in perigonadal fat had entered a stressed state we evaluated the expression of the latency genes *dosR*, *hspX* and *lat*. Importantly, Mtb residing in the adipose fraction and in SFV expressed the *dosR*, *hspX* and *lat* genes (Fig 2D), indicating activation of the stress-related program. The variability in the extent of the gene responses between adipose fraction and SVF could indicate different metabolic adaptations of Mtb present in those tissues. PCR reaction directed at the Mtb-specific IS6110 insertion sequence confirmed the presence of Mtb in adipose tissue from aerosol-infected mice and revealed again considerable variations between individual animals (Fig 2E). Similar to experiments in which Mtb from perigonadal fat was enumerated, in these experiments on average approximately 50% of samples were positive for Mtb. Confocal microscopy of perigonadal fat from i.v. infected mice consistently confirmed Mtb residence inside adipose tissue in vivo (Fig 2F).

To further validate the presence of Mtb in adipose tissue of mice we infected prospective donor animals i.v. Fourteen days after infection we collected the perigonadal fat, washed it extensively to avoid blood contamination, homogenized it and injected it subcutaneously to naïve recipient mice. Mtb was detected in lung, spleen, perigonadal and subcutaneous fat from previously naïve recipient mice confirming that Mtb had been present in adipose tissue of donor animals (Fig 2G). Mtb was not only found in the spleen and lung of donor mice (S1C Fig) but also in perigonadal fat of control mice infected at the same time as donors (S1D Fig).

### Leukocytes infiltrate perigonadal fat after aerogenic Mtb infection

**Fig 3:**
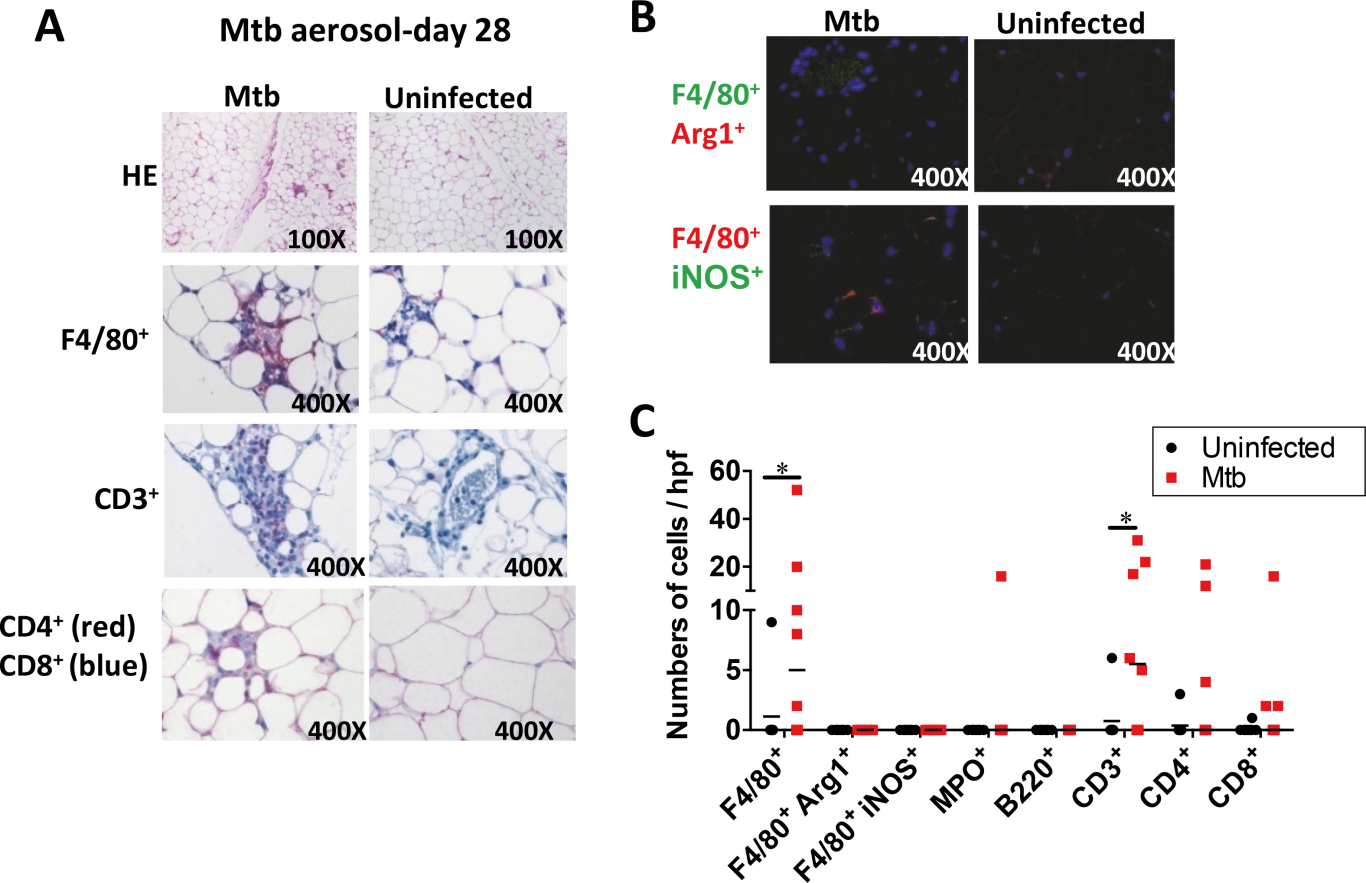
Leukocytes infiltrate perigonadal fat post aerosol-infection with Mtb. (**A**) Immunohistochemical staining for leukocyte infiltration at day 28 post aerosol infection. Data representative of two independent experiments. (**B**) Immunofluorescence staining of F4/80^+^ Arg1^+^ or iNOS^+^ cells at day 28 post aerosol- infection. Blue: DAPI. Data representative of two independent experiments. (**C**) Number of leukocytes per hpf at day 28 post aerosol-infection. Results of two independent experiments pooled (medians); *p<0.05 (Mann–Whitney test). Abbreviations: HE, hematoxilin and eosin staining; hpf, high power field.

Adipose tissue composition varies depending on metabolic changes. However, little is known about alterations in adipose tissue during infectious diseases including TB. Immunohistologic examination at day 28 post-aerosol infection revealed that F4/80^+^ macrophages, CD3^+^, CD4^+^ and CD8^+^ cells localized in perigonadal fat (Fig 3A and 3C), whereas no positive staining for myeloperoxidase (MPO) abundantly expressed in neutrophils, or for the pan B cell marker B220 was observed (Fig 3C). Surprisingly, neither arginase 1 (Arg1) (M2 type) nor inducible nitric oxide synthase (iNOS) (M1 type) could be detected in F4/80^+^ macrophages infiltrating perigonadal fat (Fig 3B and 3C) indicating that other cell types expressed these enzymes in adipose tissue of infected animals. The higher numbers of F4/80^+^ macrophages present in perigonadal fat after aerosol infection point to these cells as likely hosts of Mtb in the SFV as depicted in Fig 2C. Additionally, perigonadal fat tissue from Mtb-infected mice exhibited a marked staining for CD3 indicating the presence of T cells and both CD4^+^ and CD8^+^ T cell subsets outnumbered those in uninfected tissue (Fig 3A and 3C). At day 14 post-infection, no signs of leukocyte infiltration were noted. Immunohistology of infected tissue also revealed that adipocyte size did not change after infection (S2A Fig), which is in agreement with the absence of higher abundance of free fatty acids in serum of infected animals (S2B Fig). In summary, different leukocyte populations infiltrate perigonadal fat after aerogenic Mtb infection indicating phenotypic alterations in this tissue.

### Mtb infection induces changes in gene expression in perigonadal fat

**Fig 4:**
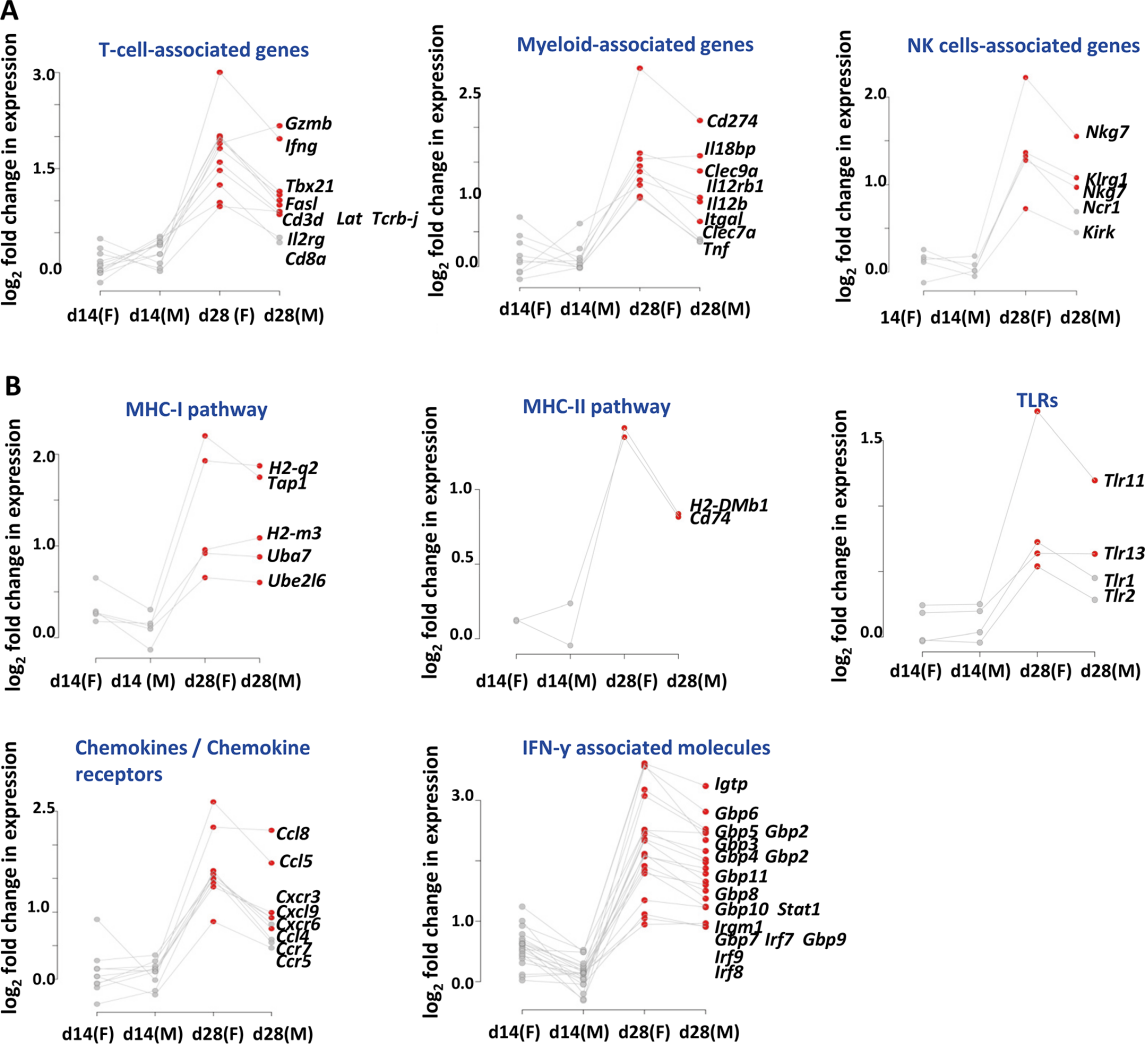
Differential gene expression in perigonadal fat post aerosol-infection with Mtb. (**A-B**) Changes in gene expression in perigonadal fat at days 14 and 28 post-infection. Grey dots represent non-significant mean log_2_ fold increments from uninfected mice (six samples per group). Red dots represent significant mean log_2_ fold increments from uninfected mice (six samples per group). (**A**) Cell type-associated changes in gene expression. (**B**) Pathway-associated changes in gene expression. Data representative of two independent experiments. Abbreviations: d, day; F, female; M, male.

We determined the global gene expression profile of perigonadal adipose tissue after Mtb aerosol infection. The global transcription profile did not reveal statistically significant changes at day 14 post-infection, (Fig 4A and 4B) whereas at day 28, statistically significant differences in gene expression were observed (Fig 4A and 4B). Several differentially regulated genes support the presence of CD8^+^ T cells in perigonadal fat of infected mice (Fig 4A), including *Cd3d*, *Cd8a*, *Ifng*, *Gzmb*, *Fasl*, *Il2rg*, *Lat*, *Tbx21* and *Tcrb-j*. Increased transcription of *Il12rb1*, *Itgal*, *Cd274*, *Clec9a*, *Clec7*, *Il18bp* and *Tnf* suggested a myeloid signature and the upregulated genes *Nkg7*, *Nrc1*, *Klrg1* and *Klrk1* are characteristic of the NK cell lineage (Fig 4A). In addition, MHC-I and MHC-II (*Tap1*, *H2-q2*, *H2-m3*, *Uba7*, *Ube2l6*, *H2-dmb1*, *Cd74*), Toll-like receptor (TLR) pathways and chemokines/chemokine receptors were also differentially regulated (Fig 4B) including *Ccl5*, *Ccl8*, *Ccl4*, *Cxcl9*, *Ccr5*, *Ccr7*, *Cxcr3* and *Cxcr6*. The most highly upregulated genes comprised IFN-γ-regulated genes such as *Stat1*, *Irf9*, *Igtp*, *Irgm1* and *Gbp6* (Fig 4B). Since differential metabolism of adipose tissue between males and females has been reported [29], female and male mice were analysed separately. Even though responses were comparable between genders, males and females expressed comparable transcription profiles in qualitative terms, with females showing slightly stronger responses (Fig 4A and 4B). Taken together the gene expression profile of perigonadal fat in response to Mtb aerosol infection revealed marked prevalence of T cell activation-associated genes (Fig 4A).

**Fig 5:**
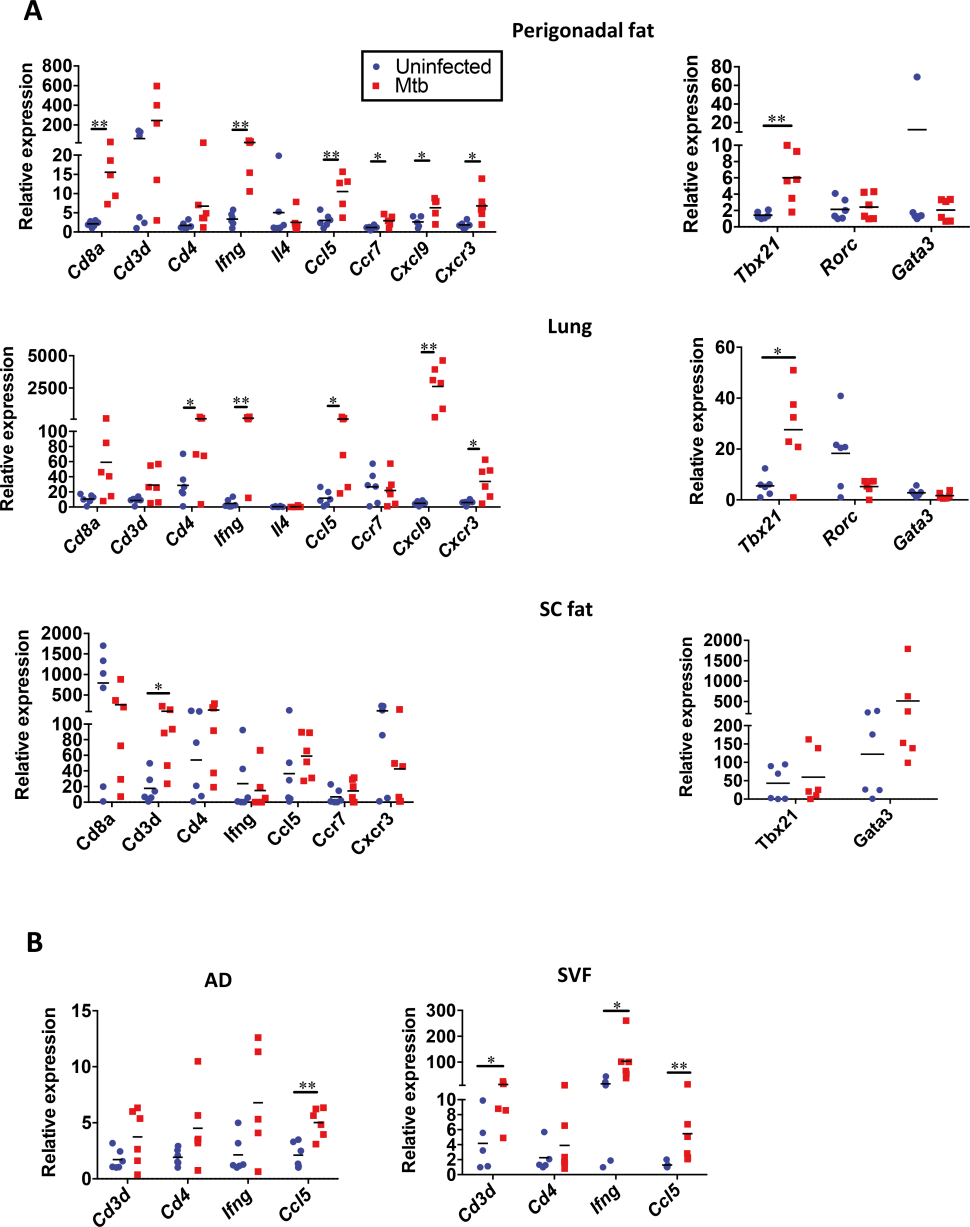
Genes associated with T cells, cytokines and chemokines are differentially upregulated in perigonadal and subcoutaneous fat post aerosol-infection with Mtb. (**A**) Expression of *Cd8a*, *Cd3d*, *Cd4*, *Ifng*, *Il4*, *Ccl5*, *Ccr7*, *Cxcl9*, *Cxcr3* (left panel) and *Tbx21*, *Rorc* and *Gata3* (right panel) in perigonadal fat, lung and subcutaneous (SC) fat, as measured with quantitative PCR at day 28 post infection. (**B**) Expression of genes as in (**A**) in AD or SVF fractions of perigonadal fat at day 28 post infection. Gene expression is relative to the lower values detected and each group is compared to their own uninfected controls. Data are representative of two to three independent experiments (means); *p<0.05 and **p<0.01 (Student’s t-test). Abbreviations: AD, adipose fraction; SC, subcutaneous; SVF, stromal vascular fraction.

To validate these results, qPCR analyses of selected genes were performed in samples from female mice. No significant changes were observed in perigonadal fat and in the lung at day 14 post-infection with the exception of IFN-γ in the latter organ (S3A Fig). In contrast, at day 28, genes associated with T cells, including *Cd8a*, *Ccr7* and *Tbx21* were highly upregulated in perigonadal fat of Mtb-infected mice together with cytokines and chemokines, including genes encoding IFN-γ, CCL5 and CXCL9 and the chemokine receptor CXCR3 (Fig 5A). Gene expression pattern in perigonadal fat at day 28 post-infection was distinct from subcutaneous fat where only *Cd3d* was upregulated (Fig 5A). This profile also diverged from that observed in lungs of infected mice, where *Cd4*, rather than *Cd8a*, was highly expressed while *Ccr7* remained unaltered (Fig 5A). We extended our analysis to later time points post-infection with Mtb. Expression of *Cd3d*, *Cd8a*, *Ifn*, *Ccl5* and *Tbx21* remained highly upregulated 56 days post-infection whereas *Ccr7* did not (S3B Fig). A similar pattern was observed in the lung where in contrast to perigonadal fat *Cd4* was also upregulated (S3B Fig).

Finally, we characterized the gene expression of adipocytes and SVF separately. As expected, the SVF was enriched in leukocyte-associated genes (Fig 5B) while only *Ccl5* was enriched in the adipocyte fraction of Mtb-infected mice (Fig 5B). Thus, changes in gene expression in perigonadal fat induced by Mtb were primarily a consequence of leukocyte infiltration and this infiltration was tissue-specific since subcutaneous fat or lung showed a different gene expression pattern. Even though Mtb was not detected in adipose tissue of all infected mice (Fig 2), gene expression patterns in perigonadal fat after infection was similar in all animals (Fig 5).

### Mtb-specific CD8^+^ T cells and IFN-γ-producing NK cells are present in perigonadal fat post-infection

For an in-depth characterization of leukocytes infiltrating perigonadal fat, we performed FACS analysis of the SVF at different time points after Mtb aerogenic infection. At day 14 post-infection, numbers of CD4^+^ and CD8^+^ T cells in perigonadal fat remained unaltered in contrast to lung tissue where higher numbers of both populations were identified (S4 Fig). Rare CD8^+^ CD44^+^ TB10.4^+^ T cells specific for a representative Mtb antigen were not detected in any of the tissues (S4 Fig) consistent with the notion that antigen-specific T cells at this time point do not accumulate in the lung in sufficient numbers to limit Mtb replication [30]. Similarly, at day 14 post-infection, numbers of CD4^+^, CD8^+^ or TCRβ^–^ NK1.1^+^ (NK) cells producing IFN-γ in the perigonadal fat or lung remained unaltered (S5 and S6 Figs). Numbers of IL-4-producing cells did not change in perigonadal fat during infection whereas in the lung the number of IL-4-producing NK cells was reduced (S5 and S6 Figs).

**Fig 6:**
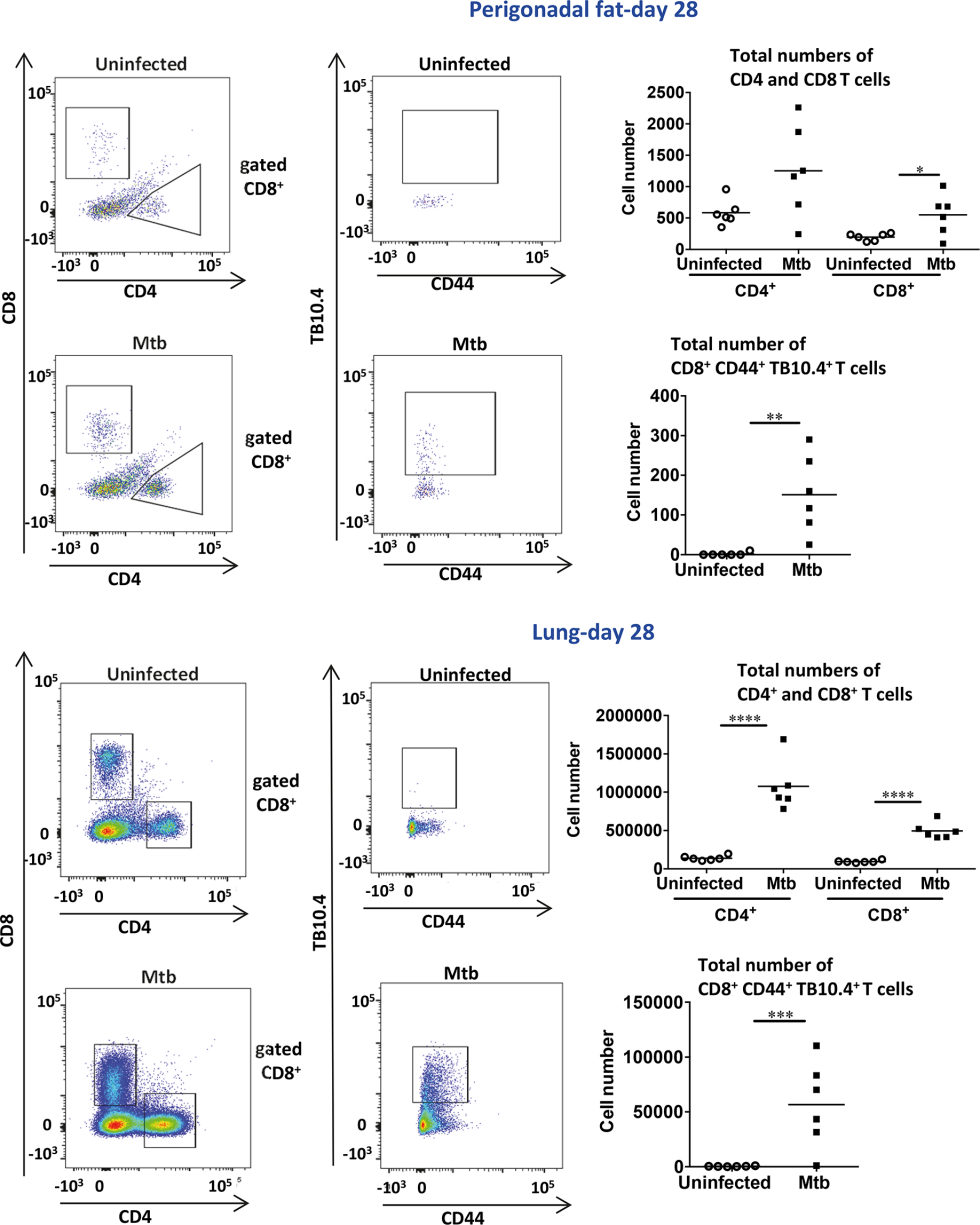
Mtb-specific CD8^+^ T cells are present in perigonadal fat post aerosol-infection. Numbers of CD4^+^, CD8^+^ and CD8^+^ CD44^+^ TB10.4^+^ (Mtb-specific) populations in SVF of perigonadal fat (upper panel) and lung (lower panel) at day 28 post infection. Data are representative of two independent experiments (means); *p<0.05, **p<0.01 and ****p<0.0001 (Student´s t-test). Abbreviations: SVF, stromal vascular fraction.

**Fig 7:**
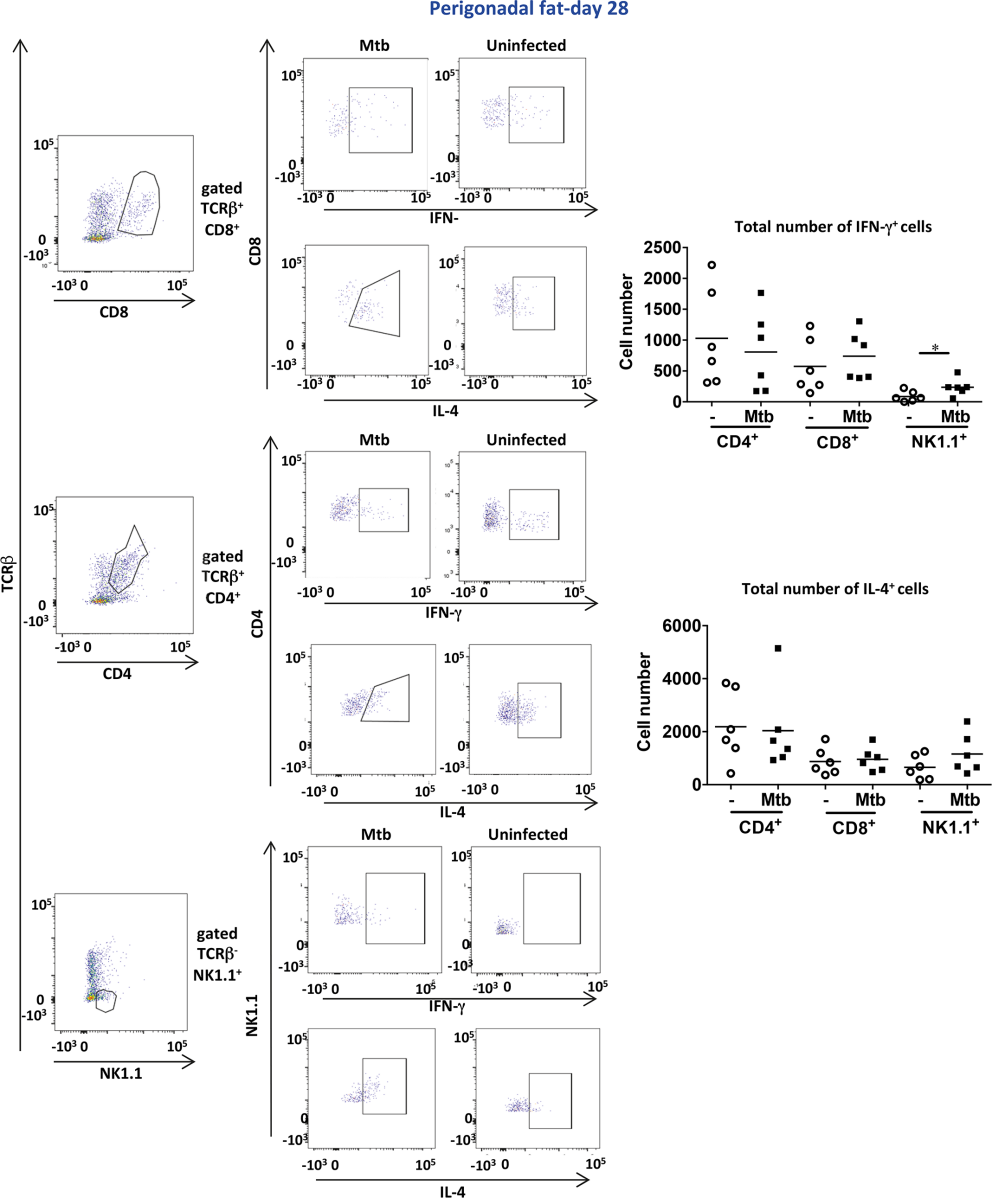
IFN-γ-producing NK cells are present in perigonadal fat post aerosol- infection. Numbers of CD4^+^, CD8^+^ and NK IFN-γ or IL-4-producing cells at day 28 post infection in SVF of perigonadal fat. Data are representative of two independent experiments (means); *p<0.05 (Student´s t-test). Abbreviations: SVF, stromal vascular fraction.

In agreement with the gene expression analysis, at day 28 post-infection, the number of total CD8^+^ T cells as well as Mtb-specific CD8^+^ T cells (CD8^+^ CD44^+^ TB10.4^+^) in perigonadal fat increased while the number of CD4^+^ T cells remained unaltered in contrast to the lung (Fig 6). Mtb-specific CD8^+^ T cells represented an average of 25.5% of the total CD8^+^ T cell population (25.5 ± 6.9%, n = 12) indicating that perigonadal fat became highly enriched in Mtb-specific CD8^+^ T cells. During infection, CD8^+^ T cells in perigonadal fat expressed an effector phenotype (CD8^+^ CD44^–^ CD69^+^) that was also seen in the lung where, in addition, CD4^+^ T cells with similar characteristics were identified (S7 Fig). It is known that the αE integrin CD103 is expressed in pathogen-specific CD8^+^ T cells in peripheral tissues [31]; here a cell population of CD8^+^ CD44^–^ CD103^+^ phenotype was identified in perigonadal fat but not in lung of infected mice (S8 Fig). FACS analysis identified NK cells as major source of IFN-γ (Fig 7). In contrast, in the lungs, CD4^+^ and CD8^+^ T cells were major IFN-γ producers in addition to NK cells (S9 Fig), and these populations also produced IL-4. In summary, at day 28 post-infection, perigonadal fat from Mtb-infected mice harbored higher numbers of total CD8^+^ T cells displaying an activated phenotype as well as Mtb-specific CD8^+^ T cells and IFN-γ-producing NK cells.

### Mtb infection modulates gene expression of Mtb-specific CD8^+^ T cells and NK cells in perigonadal fat

**Fig 8:**
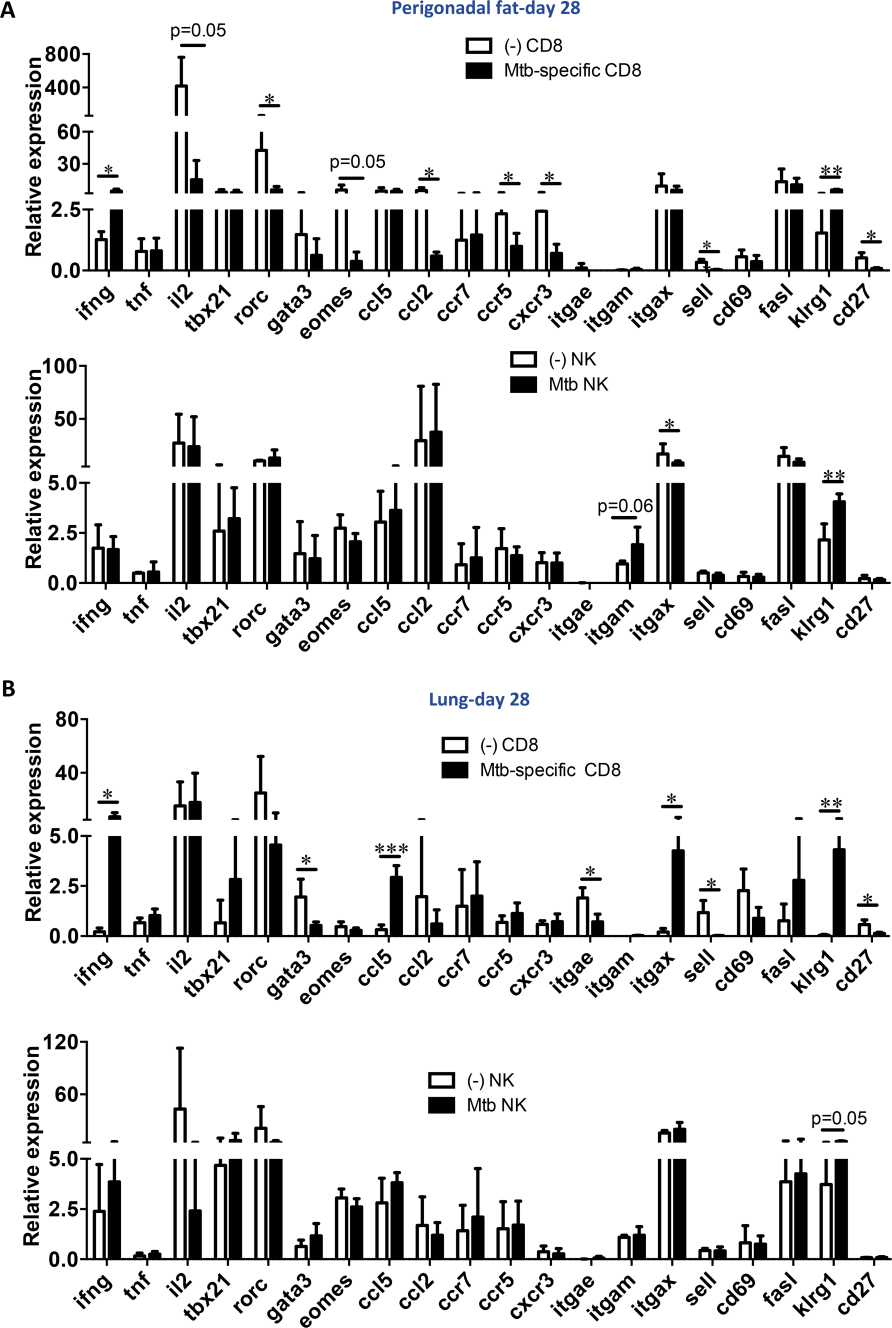
Mtb aerosol infection modulates gene expression of Mtb-specific CD8^+^ T cells and NK cells in perigonadal fat. Relative gene expression in CD8^+^ T cells from uninfected mice, Mtb-specific CD8^+^ T cells (CD8^+^ CD44^+^ TB10.4^+^) and NK cells sorted from (**A**) perigonadal fat or (**B**) lung at day 28 post-infection. Results of two independent experiments pooled (means); *p<0.05, **p<0.01 and ***p<0.001 (Student’s t-test). Abbreviations: (-), uninfected.

At day 28 post-aerosol infection, Mtb-specific CD8^+^ T cells and NK cells from perigonadal fat were sorted and selected genes were evaluated simultaneously (S10A and S10B Fig). Sorted total CD8^+^ T cells and NK cells were sorted from uninfected mice served as controls. The TB10.4 tetramer was selected as a representative antigen for sorting infiltrating Mtb-specific CD8^+^ T cells from infected mice [32]. We are aware that other Mtb specificities were still to be expected in the CD8^+^ TB10.4^-^ population. Therefore, we opted to compare CD8^+^ TB10.4^+^ cells from infected mice to total CD8^+^ cells from uninfected ones. TB10.4-specific CD8^+^ T cells from Mtb-infected mice expressed higher levels of *Ifng* than total CD8^+^ T cells from uninfected mice while the levels of *Tnf*, (Fig 8A) and *Tgfb1* (S10C Fig) were unaltered. In contrast, expression of the cytokines *Il10* and *Il17a* in TB10.4-specific CD8^+^ T cells, total CD8^+^ T cells of uninfected mice and NK cells from perigonadal fat were low (S10A Fig). Among transcription factors evaluated, TB10.4-specific CD8^+^ T cells from perigonadal fat expressed lower abundance of *Rorc* and *Eomes* than CD8^+^ T cells from uninfected mice (Fig 8A) whereas in the lungs these cells showed lower *Gata3* levels instead (Fig 8B). No significant differences were observed for *Tbx21* (T-bet) in TB10.4-specific CD8^+^ T cells and NK cells from infected mice in perigonadal fat or lung possibly due to high variations between samples (Fig 8A and 8B). Microarray analyses revealed that several chemokine and chemokine receptor genes were differentially regulated in perigonadal fat upon infection (Fig 4B). Expression levels of the *Ccl5* transcript in total CD8^+^ and TB10.4-specific CD8^+^ T cells of perigonadal fat were comparable (Fig 8A). In contrast, in the lung TB10.4-specific CD8^+^ T cells expressed a higher abundance of *Ccl5* than total CD8^+^ T cells from uninfected animals (Fig 8B). *Ccr5* and *Cxcr3* expression was also lower in TB10.4-specific CD8^+^ T cells from perigonadal fat and *Ccr7* remained unaffected after infection (Fig 8A). In the lung, the levels of *Cxcr6* were marginally higher in TB10.4-specific CD8^+^ T cells (S10D Fig). Surface expression of CD62L is down-regulated on T cells after antigen-specific activation [33]. Accordingly, Mtb-specific CD8^+^ T cells from perigonadal fat and lung exhibited lower levels of the CD62L transcript (*Sell*) than total CD8^+^ T cells from uninfected mice. Consistent with an effector rather than a memory phenotype, TB10.4-specific CD8^+^ T cells in perigonadal fat and lung showed lower levels of transcription of *Sell* (CD62L) than cells from uninfected mice (Fig 8A and 8B). On the other hand, *Cd69*, *Fasl* (Fig 8A and 8B), *Cd44*, and *Icos* (S10C and S10D Fig) were not affected while the MHC-I family member *H2-q9* expressed lower levels of transcription in cells from perigonadal fat (S10C Fig). Transcripts of the IFN-γ-induced molecules *Irgm1*, *Irf9* and *Tap1*, as well as *Zap70*, *Ube4b* and *Nkg7* were not affected by Mtb infection in TB10.4-specific CD8^+^ T cells (S10C Fig).

Killer cell lectin-like receptor subfamily G member 1 (KLRG-1) is an inhibitory C-type lectin expressed on NK cells and activated CD8^+^ T cells and a marker for terminally differentiated effector lymphocytes [34]. Both TB10.4-specific CD8^+^ T cells and NK cells in perigonadal fat showed higher abundance of *Klrg1* after Mtb infection (Fig 8A). In addition, NK cells from perigonadal fat upon Mtb infection exhibited higher levels of the integrin *Itgam* (CD11b) (Fig 8A). The appearance of CD11b in murine NK cells corresponds to progressive acquisition of effector functions [35]. Thus, at day 28 post-aerosol infection perigonadal fat tissue was enriched in TB10.4-specific CD8^+^ effector T cells and activated NK cells.

## Discussion

Our study reveals that Mtb enters and persists in adipose tissue where it transitions into a stress mode, and induces influx of NK cells and Mtb-specific effector CD8^+^ T cells to the site of its residence. We take our findings as evidence that adipose tissue serves as a potential sanctuary for persistent Mtb.

Several pathogens exploit adipose tissue for persistence. For example, *P*. *berghei* accumulates in mouse adipose tissue in form of schizont-infected erythrocytes [36] and in malaria patients the presence of *P*. *falciparum* in adipose tissue is apparently related to parasite survival [37]. *T*. *cruzi*, the causative agent of Chagas disease was also detected in biopsies of some infected individuals [7]. Similarly, in our experiments Mtb was identified in adipose tissue of approximately half of aerosol-infected mice and the pathogen was localized in both adipocytes and SVF. It is tempting to speculate that Mtb finds suitable conditions for persistence in adipocytes due to the higher abundance of triacylglycerol in adipocyte lipid droplets, which is critical for onset of dormancy in Mtb [38]. Human and murine adipocytes differed in the stressed-related genes upregulated by Mtb upon infection probably reflecting differences in host-pathogen interactions due to differences between species [39]. Not mutually exclusive, macrophages present in SVF are also a potential host for Mtb. Our experiments show that adipose-tissue resident Mtb is able to colonize lung, spleen and perigonadal fat when transferred to naïve animals pointing out that Mtb is viable despite its stress-related status. Further studies precisely characterizing the gene expression profile of Mtb in adipose tissue are needed for deeper understanding of metabolic changes occurring in the mycobacteria.

Our experiments reveal marked impact of Mtb on immune surveillance including leukocyte infiltration in adipose tissue. *Nippostrongylus brasiliensis* infection affects adipose tissue metabolism by increasing the number of eosinophils in perigonadal fat [40]. Simian immunodeficiency virus (SIV) in adipose tissue of macaques causes influx of leukocytes and activated CD4^+^ T cells have been identified in adipose tissue from HIV^+^ individuals [13]. In our study F4/80^+^ macrophages and T cells infiltrated adipose tissue by day 28 of aerogenic Mtb infection while no infiltration was detected at day 14 reflecting the onset of the adaptive immune response by day 21 [41, 42]. At day 28 almost 25% of the CD8^+^ T cells infiltrating perigonadal fat were Mtb-specific indicating that these cells are specifically attracted to the tissue after infection. Leukocyte infiltration into adipose tissue has been mostly studied in relation to obesity rather than infection [43, 44]. Here we characterized the infiltration of adipose tissue of lean mice after infection. Whether the same pattern is observed in obese mice remains to be clarified. Such studies will help provide deeper insights into the relationship between obesity, type 2 diabetes and TB.

Global gene expression and fluorescence-activated cell sorting analyses provided compelling evidence for NK and Mtb-specific CD8^+^ T cells infiltration as well as IFN-γ induction at day 28 post-infection. T cells enter adipose tissue after high fat diet [20] coincident with onset of insulin resistance [44]. In a mouse model of experimental TB, no changes in serum insulin levels were observed upon infection (uninfected: 1.70 ± 0.46 ng/ml; Mtb: 4.14 ± 2.94 ng/ml; data representative of 3 independent experiments; 4 to 5 animals per group) indicating that CD8^+^ T cells do not induce insulin resistance. We assume that Mtb-specific CD8^+^ T cells likely control intracellular Mtb in adipose tissue by virtue of lysis of Mtb-infected cells and IFN-γ secretion [45]. Enrichment of CXCR6 Mtb-specific CD8^+^ T cells in lung, which correlates with protective immunity [46] is consistent with this assumption. A similar role for IFN-γ-producing NK cells can be inferred since these cells numerically increase in the lung after infection [47] and are also able to lyse infected cells [48, 49]. Finally, IFN-γ has been associated with phenotypic polarization towards pro-inflammatory M1 cells in adipose tissue in obesity [19] and IFN-γ-producing NK cells have been linked to insulin resistance [50]. Local production of IFN-γ activates macrophages and T cells which, in turn, upregulate IFN-γ-regulated molecules. These include STAT1 and Irgm1 together with interferon-induced Gbp1 and Gbp5 and distinct chemokines including CXCL9, which amplify the inflammatory response through a feed-forward loop, resulting in chronic immune activation in TB. *Gbp1*, *Stat1* and *Tap1*, which are part of a biosignature for subclinical TB in humans [51] were enriched in perigonadal fat during Mtb infection. CD8^+^ adipose tissue infiltrating cells expressed CD69 and downregulated CD62L transcripts suggesting that these activated cells contribute to control of Mtb in fat tissue. Interestingly, after Mtb infection we identified a unique population of CD8^+^ T cells present in perigonadal fat that was absent in the lung: CD8^+^ CD44^–^ CD103^+^. This population plays a balancing role in an inflammatory model of chronic murine ileitis [52] and the αE integrin CD103 is expressed by pathogen-specific CD8^+^ T cells in peripheral tissues [31]. We therefore feel confident that CD8^+^ T cells in adipose tissue contribute to regional control of Mtb and regulation of inflammation.

In sum, this is the first report to describe persistence of Mtb expressing stress-related genes in adipocytes and the recruitment of activated immune cells to adipose tissue. We also demonstrate that Mtb present in adipose tissue can be transferred to naïve animals. Together, our findings point to adipose tissue as a potential reservoir for persistent Mtb. Better understanding of the role of adipose tissue in Mtb infection will provide the basis for rational intervention measures directed at comorbidity of TB and type 2 diabetes for which strong epidemiological evidence exists [14–18].

## Methods

### Ethic statements

Human adipocytes were obtained from plastic surgery waste tissue from patients of the Department of General, Visceral, Vascular and Thoracic Surgery (Sub-area: Plastic and Reconstructive Surgery), Charité University Medical Center in Berlin according to the regulations of and approval by the Ethics Committee of Charité, University Medical Center, Berlin, Germany (reference number EA1/249/11). All human samples were anonymized. Animal procedures were performed in accordance with the German “Tierschutzgesetz in der Fassung vom 18. Mai 2006 (BGBI.IS.1207)” and the guideline 2010/63/EU from the European Union and the European Convention for the protection of vertebrate animals used for experimental and other scientific purposes. Animal protocols were approved by the ethics committee and the Berlin state authorities (LAGeSo, reference number G179/12).

### Cell cultures and Mtb infection

The preadipocyte human cell strain Simpson-Golabi-Behmel syndrome (SGBS), kindly provided by Prof. M. Wabitsch (Department of Pediatrics and Adolescent Medicine, University of Ulm, Germany), was cultured and differentiated as described [53]. Human adipose tissue (see *Ethic statements*) was separated from skin, minced and digested with type I collagenase (Worthington Chemicals) at 200 U/ml and 3 ml/g in HBSS for 1 h at 37°C under shaking. Samples were centrifuged at 400 *g* for 10 min, erythrocytes in the pellet were lysed and cells were filtered through 70 μm and 40 μm cell strainers. After a density gradient (Biochrome) separation, the interface was collected and cell depletion performed with anti-CD45 (5B1; Miltenyi), anti-CD31 antibodies (WM59; Serotec) and anti-fluorescein isothiocyanate (FITC) beads (Miltenyi). Cells were cultured in Dulbecco’s modified Eagle medium (DMEM):F12 (Gibco) 50% heat-inactivated fetal calf serum (FCS; Gibco) and then in 20% serum for differentiation as described [53]. Murine 3T3-L1 cell line was grown in DMEM 10% FCS (Gibco), 4 mM L-glutamine (Biochrome), 1 mM sodium pyruvate (Biochrome), and 1.5 g/L sodium bicarbonate (Gibco) until confluency. Differentiation was initiated by addition of 1 μg/ml bovine insulin, 0.5 mM 3-isobutyl-1-methylxanthine (IBMX), and 1 μM dexamethasone (all from Sigma). At day 4, medium was replaced with medium containing 1 μg/ml of bovine insulin only and differentiation was complete at day 8. Cells were infected with Mtb H37Rv at a multiplicity of infection (MOI) of 5. Bacteria were grown in Middlebrook 7H9 broth (BD Biosciences) supplemented with 0.05% glycerol, Tween 80 and 10% albumin dextrose catalase (ADC) growth supplement (BD Biosciences). Single bacteria resuspended in culture medium were obtained from early log-phase cultures. After 2 h of infection, 200 μg/ml of amikacin (Sigma) was added. After 4 h, cells were washed. At different time points, cells were lysed with 0.1% Triton X-100 (ICN Biomedicals) and plated on Middlebrook 7H10 agar plates. Mtb colonies were enumerated after 3 to 6 weeks of incubation at 37°C.

### Immunofluorescence staining

Human or murine adipocytes were infected with Mtb-GFP at MOI 20 for 24 h. Mtb was detected with an anti-Mtb antibody (Abcam) and secondary antibody conjugated with Alexa 546 (Invitrogen). Nuclei were stained with 4’,6-diamidino-2-phenylindole (DAPI). Small pieces of paraformaldehyde (PFA)-fixed Mtb-infected adipose tissue were embedded in low melting point agarose (3.3% in PBS). The agarose was gelled on ice and the block containing the adipose tissue was sectioned at 300 μm thickness using a Leica VT 1000 S vibratome. Sections were stained with the DNA-intercalating dye Draq5, mounted and analyzed using a Leica TCS5 confocal microscope.

### Mice and Mtb infection

Female and male 8- to 10-week-old C57BL/6 mice were kept under specific pathogen-free conditions at the Max Planck Institute for Infection Biology in Berlin, Germany (see *Ethic statements*). Mtb strain H37Rv was grown in Middlebrook 7H9 broth (BD Biosciences) supplemented with 0.2% glycerol, 0.05% Tween 80, and 10% ADC enrichment (BD Biosciences) until logarithmic growth phase before storage at –80°C. Animals were aerosol-infected with 50–200 colony-forming units (CFUs) Mtb, using a Glas-Col inhalation exposure system. At given time points, serial dilutions of lung, spleen or undiluted perigonadal fat homogenates were plated onto Middlebrook 7H11 plates. CFUs were counted after 3 (lungs and spleen) or 6 (perigonadal fat) weeks of incubation at 37°C.

### Detection of mycobacterial DNA

PCR was performed by incubation of the DNA samples with the IS6110 Mtb insertion sequence primers 5’-CGTGAGGGCATCGAGGTGGC-3’ and 5’-GCGTAGGCGTCG GTGACAAA-3’ and the Hot Star Mix (Qiagen).

### Microarray analysis

Gene expression microarray studies were carried out with the SurePrint G3 Mouse GE 8×60K Microarray Kit (Agilent Technologies, product number G4852A). Microarray data were deposited in the NCBI’s Gene Expression Omnibus (GEO accession number GSE83554). Microarrays were background corrected, normalized and statistically analysed with limma [54] with moderated t-test for significance of the factors (sex and experimental group) and the interaction between sex and treatments for both day 14 and day 28 samples. P-values were corrected for multiple comparisons with the Benjamini-Hochberg method. Genes were tested for enrichment in functional associations using the R package tmod [55] with the CERNO test with Benjamini-Hochberg correction. Detailed R scripts used in the analysis are available upon request.

### Perigonadal fat, lung cell isolation, flow cytometry, and cell sorting

Perigonadal adipose tissue was placed in DMEM (Gibco) with 20 mM HEPES (Gibco) and 10 mg/ml free fatty acid–bovine serum albumin (FFA-BSA; Sigma) and minced to fine pieces. Samples were extensively washed to eliminate all traces of blood and incubated with 280 U/ml collagenase type I (Worthington), and 50 U/ml DNAse (Roche) for 45 min under shaking and passed through a 250 μm mesh (Pierce). Cells were centrifuged at 1,000 *g* for 10 min, and resuspended in PBS, 5% EDTA, 0.2% FFA-BSA. Single-cell suspensions from lungs of mice were prepared as previously described [56]. Immune cells were stained with antibodies against T cell receptor-beta chain (TCRβ) (H57-597; BD Biosciences), CD4 (RM4-5; BD Biosciences), CD8 (56–6.7; BD Biosciences), CD44 (IM7), CD69 (H1.2F3; BD Biosciences), CD103 (2E7; eBioscience), NK1.1 (PK136; eBioscience), CD62L (MEL-14; eBioscience), IFNγ (XMG1.2; BD), and IL-4 (11B11; eBioscience). H-2Kb:TB10.4 (4–11: IMYNYPAM) tetramers were prepared in-house. To stain for intracellular cytokines, cells were incubated with brefeldin A 10 μg/ml, ionomycin 1 μg/ml and phorbol 12,13-dibutyrate 50 ng/ml (all from Sigma) for 4 h at 37°C, 5% CO_2_ and permeabilized with Cytofix/Cytoperm kit (BD Biosciences) according to manufacturer’s instructions. Cells were acquired on a Canto II flow cytometer (BD Biosciences) and analyzed with FACSDiva (BD Biosciences) software. For sorting experiments cells from three animals were pooled and stained with H-2Kb:TB10.4 (4–11: IMYNYPAM) tetramers, antibodies against TCRβ (H57-597; BD Biosciences), CD4 (RM4-5; BD Biosciences), CD8 (56–6.7; BD Biosciences), CD44 (IM7) and NK1.1 (PK136; eBioscience) and sorted on a FACSAria II (BD Biosciences).

### Immunopathology

Paraffin sections were dewaxed and stained histochemically with hematoxylin and eosin (HE) for overview. For immunohistochemistry, sections were subjected to a heat-induced epitope retrieval step except for the detection of B cells prior to incubation with antibodies against CD3 (code A0452; Dako), B220 (RA3-6B2; BD Bioscience), myeloperoxidase (MPO; code 9661; Cell Signaling), CD4 (4SM95; eBioscience), or CD8 (4SM15; eBioscience). This step was followed by incubation with biotinylated secondary antibodies (Dianova). For detection, alkaline phosphatase (AP)-labelled streptavidin and chromogen RED (both Dako) were employed. For detection of macrophages, sections were subjected to protein-induced epitope retrieval employing protease (Sigma) prior to incubation with anti-F4/80 (BM8; eBioscience) followed by incubation with biotinylated rabbit anti-rat (Dako) secondary antibody. Biotin was detected using AP-labelled streptavidin (Dako) and AP was visualized with chromogen RED (Dako). For detection of classically activated (M1) macrophages the sections were subjected to a heat-induced epitope retrieval step prior to incubation with anti-inducible nitric oxide synthase (iNOS) (code ab15323; Abcam,). The EnVision+ System, HRP Labelled Polymer Anti-Rabbit (Dako) was used for detection. Nuclei were counterstained with hematoxylin (Merck). For the detection of alternatively-activated (M2) macrophages, dewaxed sections were incubated with anti-arginase 1 (N20; Santa Cruz) followed by incubation with biotinylated donkey anti-goat (Dianova). For detection AP-labelled streptavidin and chromogen RED (both Dako) were employed. After color development, sections were subjected to protein-induced epitope retrieval as described above prior to incubation with anti-F4/80 (BM8; eBioscience). Alexa488-labelled secondary antibody (Invitrogen) was used for detection. Nuclei were counterstained with DAPI (Sigma). Negative controls were performed by omitting the primary antibody. Images were acquired using the AxioImager Z1 microscope (Carl Zeiss MicroImaging). All evaluations were performed in a blinded manner.

### Quantitative real time-PCR

Perigonadal fat samples was collected in TRIzol total RNA isolation reagent (Invitrogen) and RNA was isolated as previously described [57]. For mRNA quantification, RNA was reverse-transcribed to cDNA, and qRT-PCR was performed according to manufacturer’s instructions (BioRad) TaqMan qRT-PCR assays with specific probes for mouse *Actinb* (NM_007393.4), *Cd8a* (NM_001081110.2), *Cd3d* (NM_013487.3), *Cd4* (NM_013488.2), *Ifng* (NM_008337.3), *Il4* (NM_021283.2), *Ccl5* (NM_013653.3), *Ccr7* (NM_007719.2), *Cxcl9* (NM_008599.4), *Cxcr3* (NM_009910.3), *Tbx21* (NM_019507.2), *Rorc* (NM_011281.2), *Gata3* (NM_008091.3) (Applied Biosystems) were used. TaqMan probes for the mycobacterial genes *sigA (Rv2703)*, *dosR (Rv3133c)*, *lat* (*Rv3290c*), and *hspX* (*Rv2031*) were designed by the manufacturer (Applied Biosystems). Samples from perigonadal fat were pre-amplified with TaqMan PreAmp Master Mix according to manufacturer’s protocol (Applied Biosystems). All probes were normalized to β-actin as internal control (Applied Biosystems), except for the quantification of mycobacterial genes where *sigA* was the internal control. All fold changes were calculated using the ΔΔCt method [58] and normalized to the lowest value in each group. Amplifications were performed with Step One Plus (Applied Biosystems).

Gene expression of sorted CD8^+^ T cells from uninfected mice, Mtb-specific CD8^+^ T cells from infected mice and NK cells from uninfected and infected mice were analyzed simultaneously using the 48.48 Dynamic Array Integrated Fluidic Circuits (IFCs; Fluidigm). Triplicates of 100 sorted cells were collected in a 96-well PCR plate (Eppendorf) containing CellDirect Reaction mix (Life Technologies) with Ambion SUPERase-In (Ambion) and stored at –80°C. Pre-amplification of genes by reverse transcription and cDNA synthesis (18 cycles) was performed using Cells Direct One-Step qPCR Kit (Life Technologies) and TaqMan gene expression assay mix (Applied Biosystems). The cDNA and the single TaqMan assays were then loaded in a microfluidic chip (Fluidigm) using Fluidigm 48.48 IFC Controller MX according to manufacturer's protocol and quantitative PCR was run using the Data Collection Software (36 cycles; Fluidigm). Data were exported with the Real-time PCR Analysis Software (Fluidigm) and analyzed with Microsoft Office Excel. mRNA amounts were normalized to β-actin (NM_007393.4) expression. To compare data from different animals, tissues and chips fold change (2^–[(∆Ct)reference−∆Ct(value)]^) in transcripts was calculated relative to splenic CD8^+^ T cells, which were sorted in each plate as internal reference [59]. The following transcripts were evaluated: *Cd8a* (NM_001081110.2), *Cd3d* (NM_013487.3), *Cd4* (NM_013488.2), *Ifng* (NM_008337.3), *Il4* (NM_021283.2), *Ccl5* (NM_013653.3), *Ccr7* (NM_007719.2), *Cxcl9* (NM_008599.4), *Cxcr3* (NM_009910.3), *Tbx21* (NM_019507.2), *Rorc* (NM_011281.2), *Gata3* (NM_008091.3), *Eomes* (NM_001164789.1), *Serpine1* (NM_008871.2), *Ube4b* (NM_022022.3), *Fasl* (NM_001205243.1), *Cxcr6* (NM_030712.4), *Tnf* (NM_001278601.1), *Il10* (NM_010548.2), *Il2* (NM_008366.3), *Tlr2* (NM_011905.3), *Irgm1* (NM_008326.1), *Irf9* (NM_008394.3), *Itgax* (NM_021334.2), *Tgfb1* (NM_011577.1), *Il17a* (NM_010552.3), *Itgae* (NM_008399.2), *Pdcd1* (NM_008798.2), *Tap1* (NM_001161730.1), *Nkg7* (NM_024253.4), *Foxp3* (NM_001199347.1), *Il15* (NM_008357.2), *Itgam* (NM_008401.2), *Cd27* (NM_001033126.2), *Sell* (NM_001164059.1), *Cd44* (NM_009851.2), *Ccl2* (NM_011333.3), *Ccr5* (NM_009917.5), *Zap70* (NM_009539.2), *Fcgr1* (NM_010186.5), *Icos* (NM_017480.2), *Cd69* (NM_001033122.3), *Klrg1* (NM_016970.1), *Ncr1* (NM_010746.3), *Cd5* (NM_007650.3), *H2-q7/h2-q9* (NM_001201460.1), and *Nampt* (NM_021524.2) (Applied Biosystems).

### Statistical analysis

Differences were analyzed using Student’s t-test (parametric groups) or Mann–Whitney U test (nonparametric groups). P values <0.05 were considered statistically significant.

## Supporting information

S1 Fig Mtb resides in perigonadal and subcutaneous fat post aerosol-infection of mice and in spleen and lung of donor mice and in spleen, lung and perigonadal fat of control mice in fat transfer experiments. (**A**) log_10_ Mtb CFUs in in spleen, lung and AD and SVF fractions of perigonadal fat at different time points after aerosol infection. Data representative of four independent experiments (medians). (**B**) log_10_ Mtb CFUs in perigonadal and subcoutaneous fat at day 28 post aerosol-infection (200 CFUs). Data are representative of two independent experiments (medians). (**C**) log_10_ Mtb CFUs in spleen and lung from donor mice used for transfer experiments. Mice were infected i.v. with 5x10^6^ CFUs of Mtb, organs were collected 14 days after infection and perigonadal fat was transferred to uninfected recipient mice. Data representative of two independent experiments (medians). (**D**) log_10_ Mtb CFUs in spleen, lung, and perigonadal (P) fat from control mice infected i.v with 5x10^6^ CFUs of Mtb at the same time as the mice used for transfer experiments. Organs were collected 14 days after infection. Data representative of two independent experiments (medians). Abbreviations: AD, adipose fraction; P, perigonadal; SC, subcutaneous; SVF, stromal vascular fraction.
(TIF)

S2 Fig Mtb infection does not alter adipocyte size in perigonadal fat. (**A**) Adipocyte size at day 28 post aerosol-infection. (**B**) Free fatty acids in sera at day 14 and 28 post infection. Data representative of two independent experiments. Abbreviations: FFA, free fatty acids.
(TIF)

S3 Fig Differential gene expression in perigonadal fat and lung post aerosol- infection with Mtb. (**A-B**) Expression of *Cd8a*, *Cd3d*, *Cd4*, *Ifng*, *Il4*, *Ccl5*, *Ccr7*, *Cxcl9*, *Cxcr3* (left panel) and *Tbx21*, *Rorc* and *Gata3* (right panel) in perigonadal fat and lung, as measured with quantitative PCR at: (**A**) day 14 or (**B**) day 56 post infection. Data are representative of two to three independent experiments (means); *p<0.05, **p<0.01 and ***p<0.001 (Student’s t-test).
(TIF)

S4 Fig Numbers of CD4^+^, CD8^+^, CD8^+^ CD44^+^ TB10.4^+^ (Mtb-specific) populations in SVF of perigonadal fat and lung post aerosol-infection with Mtb. Numbers of CD4^+^, CD8^+^ and CD8^+^ CD44^+^ TB10.4^+^ (Mtb-specific) populations in SVF of perigonadal fat (upper panel) and lung (lower panel) at day 14 and post infection. Data are representative of two independent experiments (means); *p<0.05 and ***p<0.001 (Student´s t-test). Abbreviations: SVF, stromal vascular fraction.
(TIF)

S5 Fig Numbers of CD4^+^, CD8^+^, and NK IFN-γ or IL-4-producing cells in SVF of perigonadal fat at day 14 post aerosol-infection with Mtb. Numbers of CD4^+^, CD8^+^ and NK IFN-γ or IL-4-producing cells at day 14 post infection. Data are representative of two independent experiments (means). Abbreviations: SVF, stromal vascular fraction.
(TIF)

S6 Fig Numbers of CD4^+^, CD8^+^, and NK IFN-γ or IL-4-producing cells in lung at day 14 post aerosol-infection with Mtb. Numbers of CD4^+^, CD8^+^ and NK IFN-γ or IL-4-producing cells at day 14 post infection. Data are representative of two independent experiments (means); *p<0.05 (Student´s t-test).
(TIF)

S7 Fig Effector CD8^+^ T cells are present in perigonadal fat post aerosol- infection. Numbers of CD4^+^ CD44^–^ CD69^+^ and CD8^+^ CD44^–^ CD69^+^ cells in SVF of perigonadal fat (upper panel) or lung (lower panel) at day 28 post infection. Data are representative of two independent experiments (means); *p<0.05, and ****p<0.0001 (Student´s t-test). Abbreviations: SVF, stromal vascular fraction.
(TIF)

S8 Fig CD8^+^ CD44^–^ CD103^+^ T cells are present in perigonadal fat post aerosol- infection. Numbers of CD4^+^ CD44^–^ CD103^+^ and CD8^+^ CD44^–^ CD103^+^ cells in SVF of perigonadal fat (upper panel) or lung (lower panel) at day 28 post infection. Data are representative of two independent experiments (means); *p<0.05, and ****p<0.0001 (Student´s t-test). Abbreviations: SVF, stromal vascular fraction.
(TIF)

S9 Fig Numbers of CD4^+^, CD8^+^, and NK IFN-γ or IL-4-producing cells in lung at day 28 post aerosol-infection with Mtb. Numbers of CD4^+^, CD8^+^ and NK IFN-γ or IL-4-producing cells in lung at day 28 post infection. Data are representative of two independent experiments (means); *p<0.05, ***p<0.001 and ****p<0.0001 (Student´s t-test).
(TIF)

S10 Fig Mtb infection modulates gene expression of Mtb-specific CD8^+^ T cells and NK cells in perigonadal fat. (**A**) Heat maps of gene expression of CD4^+^, CD8^+^, Mtb-specific CD8^+^ T cells (CD8^+^ CD44^+^ TB10.4^+^) and NK cells sorted from perigonadal fat (right panel) or lung (left panel) at day 28 post infection. Colour corresponds to ΔCt values. Mtb infection modulates gene expression in NK cells and Mtb-specific CD8^+^ T cells in perigonadal fat. (**B-D**) Relative gene expression of CD8^+^, CD8^+^ TB10.4 (Mtb-specific) and NK cells sorted from perigonadal fat (**B-C**) or lung (**D**) at day 28 post infection. Results of two independent experiments pooled (means); *p<0.05, **p<0.01 and ***p<0.001 (Student’s t-test). Abbreviations: (-), uninfected.
(TIF)

## References

[1] WHO World Health Organization G. Global Tuberculosis Report 2016. 2016.

[2] Raupach B, Kaufmann SH. Immune responses to intracellular bacteria. Curr Opin Immunol. 2001;13(4):417–28. Epub 2001/08/11. . 11498297 10.1016/s0952-7915(00)00236-3

[3] Flynn JL, Chan J. Tuberculosis: latency and reactivation. Infect Immun. 2001;69(7):4195–201. Epub 2001/06/13. doi: 10.1128/IAI.69.7.4195-4201.2001 ; PubMed Central PMCID: PMC98451. 11401954 10.1128/IAI.69.7.4195-4201.2001 PMC98451

[4] Ehlers S. Lazy, dynamic or minimally recrudescent? On the elusive nature and location of the mycobacterium responsible for latent tuberculosis. Infection. 2009;37(2):87–95. Epub 2009/03/25. doi: 10.1007/s15010-009-8450-7 . 19308316 10.1007/s15010-009-8450-7

[5] Neyrolles O, Hernandez-Pando R, Pietri-Rouxel F, Fornes P, Tailleux L, Barrios Payan JA, et al Is adipose tissue a place for Mycobacterium tuberculosis persistence? PloS one. 2006;1:e43 Epub 2006/12/22. doi: 10.1371/journal.pone.0000043 ; PubMed Central PMCID: PMC1762355. 17183672 10.1371/journal.pone.0000043 PMC1762355

[6] Desruisseaux MS, Nagajyothi, Trujillo ME, Tanowitz HB, Scherer PE. Adipocyte, adipose tissue, and infectious disease. Infect Immun. 2007;75(3):1066–78. Epub 2006/11/23. doi: 10.1128/IAI.01455-06 ; PubMed Central PMCID: PMC1828569. 17118983 10.1128/IAI.01455-06 PMC1828569

[7] Ferreira AV, Segatto M, Menezes Z, Macedo AM, Gelape C, de Oliveira Andrade L, et al Evidence for Trypanosoma cruzi in adipose tissue in human chronic Chagas disease. Microbes and infection / Institut Pasteur. 2011;13(12–13):1002–5. Epub 2011/07/06. doi: 10.1016/j.micinf.2011.06.002 . 21726660 10.1016/j.micinf.2011.06.002 PMC3552247

[8] Wu BN, O'Sullivan AJ. Sex differences in energy metabolism need to be considered with lifestyle modifications in humans. J Nutr Metab. 2011;2011:391809 Epub 2011/07/21. doi: 10.1155/2011/391809 ; PubMed Central PMCID: PMC3136178. 21773020 10.1155/2011/391809 PMC3136178

[9] Gregor MF, Hotamisligil GS. Inflammatory mechanisms in obesity. Annu Rev Immunol. 2011;29:415–45. Epub 2011/01/12. doi: 10.1146/annurev-immunol-031210-101322 . 21219177 10.1146/annurev-immunol-031210-101322

[10] Kim JS, Ryu MJ, Byun EH, Kim WS, Whang J, Min KN, et al Differential immune response of adipocytes to virulent and attenuated Mycobacterium tuberculosis. Microbes and infection / Institut Pasteur. 2011;13(14–15):1242–51. Epub 2011/08/05. doi: 10.1016/j.micinf.2011.07.002 . 21813088 10.1016/j.micinf.2011.07.002

[11] Bouwman JJ, Visseren FL, Bouter KP, Diepersloot RJ. Infection-induced inflammatory response of adipocytes in vitro. International journal of obesity. 2008;32(6):892–901. Epub 2008/03/19. doi: 10.1038/ijo.2008.36 . 18347604 10.1038/ijo.2008.36

[12] Maurin T, Saillan-Barreau C, Cousin B, Casteilla L, Doglio A, Penicaud L. Tumor necrosis factor-alpha stimulates HIV-1 production in primary culture of human adipocytes. Experimental cell research. 2005;304(2):544–51. Epub 2005/03/08. doi: 10.1016/j.yexcr.2004.12.003 . 15748898 10.1016/j.yexcr.2004.12.003

[13] Damouche A, Lazure T, Avettand-Fenoel V, Huot N, Dejucq-Rainsford N, Satie AP, et al Adipose Tissue Is a Neglected Viral Reservoir and an Inflammatory Site during Chronic HIV and SIV Infection. PLoS Pathog. 2015;11(9):e1005153 Epub 2015/09/25. doi: 10.1371/journal.ppat.1005153 ; PubMed Central PMCID: PMC4581628. 26402858 10.1371/journal.ppat.1005153 PMC4581628

[14] Lonnroth K, Williams BG, Cegielski P, Dye C. A consistent log-linear relationship between tuberculosis incidence and body mass index. Int J Epidemiol. 2010;39(1):149–55. Epub 2009/10/13. doi: 10.1093/ije/dyp308 . 19820104 10.1093/ije/dyp308

[15] Leung CC, Lam TH, Chan WM, Yew WW, Ho KS, Leung G, et al Lower risk of tuberculosis in obesity. Archives of internal medicine. 2007;167(12):1297–304. Epub 2007/06/27. doi: 10.1001/archinte.167.12.1297 . 17592104 10.1001/archinte.167.12.1297

[16] Hanrahan CF, Golub JE, Mohapi L, Tshabangu N, Modisenyane T, Chaisson RE, et al Body mass index and risk of tuberculosis and death. AIDS. 2010;24(10):1501–8. Epub 2010/05/28. doi: 10.1097/QAD.0b013e32833a2a4a ; PubMed Central PMCID: PMC3063388. 20505496 10.1097/QAD.0b013e32833a2a4a PMC3063388

[17] Feng Y, Wang F, Pan H, Qiu S, Lu J, Wu L, et al Obesity-associated gene FTO rs9939609 polymorphism in relation to the risk of tuberculosis. BMC Infect Dis. 2014;14:592 Epub 2014/11/08. doi: 10.1186/s12879-014-0592-2 ; PubMed Central PMCID: PMC4226896. 25377722 10.1186/s12879-014-0592-2 PMC4226896

[18] Dooley KE, Chaisson RE. Tuberculosis and diabetes mellitus: convergence of two epidemics. The Lancet infectious diseases. 2009;9(12):737–46. Epub 2009/11/21. doi: 10.1016/S1473-3099(09)70282-8 ; PubMed Central PMCID: PMC2945809. 19926034 10.1016/S1473-3099(09)70282-8 PMC2945809

[19] Lumeng CN, Bodzin JL, Saltiel AR. Obesity induces a phenotypic switch in adipose tissue macrophage polarization. J Clin Invest. 2007;117(1):175–84. Epub 2007/01/04. doi: 10.1172/JCI29881 ; PubMed Central PMCID: PMC1716210. 17200717 10.1172/JCI29881 PMC1716210

[20] Nishimura S, Manabe I, Nagasaki M, Eto K, Yamashita H, Ohsugi M, et al CD8+ effector T cells contribute to macrophage recruitment and adipose tissue inflammation in obesity. Nature medicine. 2009;15(8):914–20. Epub 2009/07/28. doi: 10.1038/nm.1964 . 19633658 10.1038/nm.1964

[21] Feuerer M, Herrero L, Cipolletta D, Naaz A, Wong J, Nayer A, et al Lean, but not obese, fat is enriched for a unique population of regulatory T cells that affect metabolic parameters. Nature medicine. 2009;15(8):930–9. Epub 2009/07/28. doi: 10.1038/nm.2002 ; PubMed Central PMCID: PMC3115752. 19633656 10.1038/nm.2002 PMC3115752

[22] Hernandez-Pando R, Jeyanathan M, Mengistu G, Aguilar D, Orozco H, Harboe M, et al Persistence of DNA from Mycobacterium tuberculosis in superficially normal lung tissue during latent infection. Lancet. 2000;356(9248):2133–8. Epub 2001/02/24. . 11191539 10.1016/s0140-6736(00)03493-0

[23] Boon C, Dick T. Mycobacterium bovis BCG response regulator essential for hypoxic dormancy. Journal of bacteriology. 2002;184(24):6760–7. Epub 2002/11/26. doi: 10.1128/JB.184.24.6760-6767.2002 ; PubMed Central PMCID: PMC135468. 12446625 10.1128/JB.184.24.6760-6767.2002 PMC135468

[24] Park HD, Guinn KM, Harrell MI, Liao R, Voskuil MI, Tompa M, et al Rv3133c/dosR is a transcription factor that mediates the hypoxic response of Mycobacterium tuberculosis. Molecular microbiology. 2003;48(3):833–43. Epub 2003/04/16. ; PubMed Central PMCID: PMC1992516. 12694625 10.1046/j.1365-2958.2003.03474.x PMC1992516

[25] Voskuil MI, Visconti KC, Schoolnik GK. Mycobacterium tuberculosis gene expression during adaptation to stationary phase and low-oxygen dormancy. Tuberculosis. 2004;84(3–4):218–27. Epub 2004/06/23. doi: 10.1016/j.tube.2004.02.003 . 15207491 10.1016/j.tube.2004.02.003

[26] Sukumar N, Tan S, Aldridge BB, Russell DG. Exploitation of Mycobacterium tuberculosis reporter strains to probe the impact of vaccination at sites of infection. PLoS Pathog. 2014;10(9):e1004394 doi: 10.1371/journal.ppat.1004394 ; PubMed Central PMCID: PMCPMC4169503. 25233380 10.1371/journal.ppat.1004394 PMC4169503

[27] Carey DG, Jenkins AB, Campbell LV, Freund J, Chisholm DJ. Abdominal fat and insulin resistance in normal and overweight women: Direct measurements reveal a strong relationship in subjects at both low and high risk of NIDDM. Diabetes. 1996;45(5):633–8. . 8621015 10.2337/diab.45.5.633

[28] Tchkonia T, Thomou T, Zhu Y, Karagiannides I, Pothoulakis C, Jensen MD, et al Mechanisms and metabolic implications of regional differences among fat depots. Cell Metab. 2013;17(5):644–56. doi: 10.1016/j.cmet.2013.03.008 ; PubMed Central PMCID: PMCPMC3942783. 23583168 10.1016/j.cmet.2013.03.008 PMC3942783

[29] Medrikova D, Jilkova ZM, Bardova K, Janovska P, Rossmeisl M, Kopecky J. Sex differences during the course of diet-induced obesity in mice: adipose tissue expandability and glycemic control. International journal of obesity. 2012;36(2):262–72. doi: 10.1038/ijo.2011.87 . 21540832 10.1038/ijo.2011.87

[30] Torrado E, Robinson RT, Cooper AM. Cellular response to mycobacteria: balancing protection and pathology. Trends in immunology. 2011;32(2):66–72. Epub 2011/01/11. doi: 10.1016/j.it.2010.12.001 ; PubMed Central PMCID: PMC3039081. 21216195 10.1016/j.it.2010.12.001 PMC3039081

[31] Gebhardt T, Wakim LM, Eidsmo L, Reading PC, Heath WR, Carbone FR. Memory T cells in nonlymphoid tissue that provide enhanced local immunity during infection with herpes simplex virus. Nat Immunol. 2009;10(5):524–30. doi: 10.1038/ni.1718 . 19305395 10.1038/ni.1718

[32] Skjot RL, Oettinger T, Rosenkrands I, Ravn P, Brock I, Jacobsen S, et al Comparative evaluation of low-molecular-mass proteins from Mycobacterium tuberculosis identifies members of the ESAT-6 family as immunodominant T-cell antigens. Infect Immun. 2000;68(1):214–20. ; PubMed Central PMCID: PMCPMC97123. 10603390 10.1128/iai.68.1.214-220.2000 PMC97123

[33] Sallusto F, Lenig D, Forster R, Lipp M, Lanzavecchia A. Two subsets of memory T lymphocytes with distinct homing potentials and effector functions. Nature. 1999;401(6754):708–12. doi: 10.1038/44385 . 10537110 10.1038/44385

[34] Sarkar S, Kalia V, Haining WN, Konieczny BT, Subramaniam S, Ahmed R. Functional and genomic profiling of effector CD8 T cell subsets with distinct memory fates. J Exp Med. 2008;205(3):625–40. Epub 2008/03/05. doi: 10.1084/jem.20071641 ; PubMed Central PMCID: PMC2275385. 18316415 10.1084/jem.20071641 PMC2275385

[35] Chiossone L, Chaix J, Fuseri N, Roth C, Vivier E, Walzer T. Maturation of mouse NK cells is a 4-stage developmental program. Blood. 2009;113(22):5488–96. doi: 10.1182/blood-2008-10-187179 . 19234143 10.1182/blood-2008-10-187179

[36] Franke-Fayard B, Fonager J, Braks A, Khan SM, Janse CJ. Sequestration and tissue accumulation of human malaria parasites: can we learn anything from rodent models of malaria? PLoS Pathog. 2010;6(9):e1001032 Epub 2010/10/14. doi: 10.1371/journal.ppat.1001032 ; PubMed Central PMCID: PMC2947991. 20941396 10.1371/journal.ppat.1001032 PMC2947991

[37] Seydel KB, Milner DA Jr., Kamiza SB, Molyneux ME, Taylor TE. The distribution and intensity of parasite sequestration in comatose Malawian children. The Journal of infectious diseases. 2006;194(2):208–5. Epub 2006/06/17. doi: 10.1086/505078 ; PubMed Central PMCID: PMC1515074. 16779727 10.1086/505078 PMC1515074

[38] Daniel J, Maamar H, Deb C, Sirakova TD, Kolattukudy PE. Mycobacterium tuberculosis uses host triacylglycerol to accumulate lipid droplets and acquires a dormancy-like phenotype in lipid-loaded macrophages. PLoS Pathog. 2011;7(6):e1002093 Epub 2011/07/07. doi: 10.1371/journal.ppat.1002093 ; PubMed Central PMCID: PMC3121879. 21731490 10.1371/journal.ppat.1002093 PMC3121879

[39] Lin S, Lin Y, Nery JR, Urich MA, Breschi A, Davis CA, et al Comparison of the transcriptional landscapes between human and mouse tissues. Proc Natl Acad Sci U S A. 2014;111(48):17224–9. doi: 10.1073/pnas.1413624111 ; PubMed Central PMCID: PMCPMC4260565. 25413365 10.1073/pnas.1413624111 PMC4260565

[40] Wu D, Molofsky AB, Liang HE, Ricardo-Gonzalez RR, Jouihan HA, Bando JK, et al Eosinophils sustain adipose alternatively activated macrophages associated with glucose homeostasis. Science. 2011;332(6026):243–7. Epub 2011/03/26. doi: 10.1126/science.1201475 ; PubMed Central PMCID: PMC3144160. 21436399 10.1126/science.1201475 PMC3144160

[41] Chackerian AA, Alt JM, Perera TV, Dascher CC, Behar SM. Dissemination of Mycobacterium tuberculosis is influenced by host factors and precedes the initiation of T-cell immunity. Infect Immun. 2002;70(8):4501–9. Epub 2002/07/16. doi: 10.1128/IAI.70.8.4501-4509.2002 ; PubMed Central PMCID: PMC128141. 12117962 10.1128/IAI.70.8.4501-4509.2002 PMC128141

[42] Wolf AJ, Desvignes L, Linas B, Banaiee N, Tamura T, Takatsu K, et al Initiation of the adaptive immune response to Mycobacterium tuberculosis depends on antigen production in the local lymph node, not the lungs. J Exp Med. 2008;205(1):105–15. Epub 2007/12/26. doi: 10.1084/jem.20071367 ; PubMed Central PMCID: PMC2234384. 18158321 10.1084/jem.20071367 PMC2234384

[43] Schipper HS, Prakken B, Kalkhoven E, Boes M. Adipose tissue-resident immune cells: key players in immunometabolism. Trends in endocrinology and metabolism: TEM. 2012;23(8):407–15. Epub 2012/07/17. doi: 10.1016/j.tem.2012.05.011 . 22795937 10.1016/j.tem.2012.05.011

[44] Kintscher U, Hartge M, Hess K, Foryst-Ludwig A, Clemenz M, Wabitsch M, et al T-lymphocyte infiltration in visceral adipose tissue: a primary event in adipose tissue inflammation and the development of obesity-mediated insulin resistance. Arteriosclerosis, thrombosis, and vascular biology. 2008;28(7):1304–10. Epub 2008/04/19. doi: 10.1161/ATVBAHA.108.165100 . 18420999 10.1161/ATVBAHA.108.165100

[45] Woodworth JS, Behar SM. Mycobacterium tuberculosis-specific CD8+ T cells and their role in immunity. Crit Rev Immunol. 2006;26(4):317–52. Epub 2006/11/01. ; PubMed Central PMCID: PMC3134450. 17073557 10.1615/critrevimmunol.v26.i4.30 PMC3134450

[46] Lee LN, Ronan EO, de Lara C, Franken KL, Ottenhoff TH, Tchilian EZ, et al CXCR6 is a marker for protective antigen-specific cells in the lungs after intranasal immunization against Mycobacterium tuberculosis. Infect Immun. 2011;79(8):3328–37. doi: 10.1128/IAI.01133-10 ; PubMed Central PMCID: PMCPMC3147559. 21628524 10.1128/IAI.01133-10 PMC3147559

[47] Junqueira-Kipnis AP, Kipnis A, Jamieson A, Juarrero MG, Diefenbach A, Raulet DH, et al NK cells respond to pulmonary infection with Mycobacterium tuberculosis, but play a minimal role in protection. J Immunol. 2003;171(11):6039–45. Epub 2003/11/25. . 14634116 10.4049/jimmunol.171.11.6039

[48] Esin S, Batoni G, Pardini M, Favilli F, Bottai D, Maisetta G, et al Functional characterization of human natural killer cells responding to Mycobacterium bovis bacille Calmette-Guerin. Immunology. 2004;112(1):143–52. Epub 2004/04/21. doi: 10.1111/j.1365-2567.2004.01858.x ; PubMed Central PMCID: PMC1782452. 15096193 10.1111/j.1365-2567.2004.01858.x PMC1782452

[49] Stenger S, Hanson DA, Teitelbaum R, Dewan P, Niazi KR, Froelich CJ, et al An antimicrobial activity of cytolytic T cells mediated by granulysin. Science. 1998;282(5386):121–5. . 9756476 10.1126/science.282.5386.121

[50] Wensveen FM, Jelencic V, Valentic S, Sestan M, Wensveen TT, Theurich S, et al NK cells link obesity-induced adipose stress to inflammation and insulin resistance. Nat Immunol. 2015;16(4):376–85. Epub 2015/03/03. doi: 10.1038/ni.3120 . 25729921 10.1038/ni.3120

[51] Zak DE, Penn-Nicholson A, Scriba TJ, Thompson E, Suliman S, Amon LM, et al A blood RNA signature for tuberculosis disease risk: a prospective cohort study. Lancet. 2016 doi: 10.1016/S0140-6736(15)01316-1 . 27017310 10.1016/S0140-6736(15)01316-1 PMC5392204

[52] Ho J, Kurtz CC, Naganuma M, Ernst PB, Cominelli F, Rivera-Nieves J. A CD8+/CD103high T cell subset regulates TNF-mediated chronic murine ileitis. J Immunol. 2008;180(4):2573–80. ; PubMed Central PMCID: PMC3036968. 18250468 10.4049/jimmunol.180.4.2573 PMC3036968

[53] Fischer-Posovszky P, Newell FS, Wabitsch M, Tornqvist HE. Human SGBS cells—a unique tool for studies of human fat cell biology. Obesity facts. 2008;1(4):184–9. Epub 2008/01/01. doi: 10.1159/000145784 . 20054179 10.1159/000145784 PMC6452113

[54] Ritchie ME, Phipson B, Wu D, Hu Y, Law CW, Shi W, et al limma powers differential expression analyses for RNA-sequencing and microarray studies. Nucleic Acids Res. 2015;43(7):e47 doi: 10.1093/nar/gkv007 ; PubMed Central PMCID: PMCPMC4402510. 25605792 10.1093/nar/gkv007 PMC4402510

[55] Weiner 3rd J, Domaszewska T. tmod: an R package for general and multivariate enrichment analysis. PeerJ Preprints. 2016;(e2420v1).

[56] Kursar M, Koch M, Mittrucker HW, Nouailles G, Bonhagen K, Kamradt T, et al Cutting Edge: Regulatory T cells prevent efficient clearance of Mycobacterium tuberculosis. J Immunol. 2007;178(5):2661–5. Epub 2007/02/22. . 17312107 10.4049/jimmunol.178.5.2661

[57] Guan H, Yang K. RNA Isolation and Real-Time Quantitative RT-PCR In: Yang K, editor. Adipose Tissue Protocols. Methods in Molecular Biology. 456. Second ed: Humana Press; 2008 p. 259–70. 10.1007/978-1-59745-245-8_19 18516567

[58] Livak KJ, Schmittgen TD. Analysis of relative gene expression data using real-time quantitative PCR and the 2(-Delta Delta C(T)) Method. Methods. 2001;25(4):402–8. Epub 2002/02/16. doi: 10.1006/meth.2001.1262 . 11846609 10.1006/meth.2001.1262

[59] Lozza L, Farinacci M, Bechtle M, Staber M, Zedler U, Baiocchini A, et al Communication between Human Dendritic Cell Subsets in Tuberculosis: Requirements for Naive CD4(+) T Cell Stimulation. Front Immunol. 2014;5:324 doi: 10.3389/fimmu.2014.00324 ; PubMed Central PMCID: PMCPMC4094910. 25071784 10.3389/fimmu.2014.00324 PMC4094910

