# Supplementary figures and images for: Mycobacterium tuberculosis infection modulates adipose tissue biology
Source: PLoS Pathog. 2017 Oct 17;13(10):e1006676. doi: 10.1371/journal.ppat.1006676 (PMC5695609; doi:10.1371/journal.ppat.1006676)

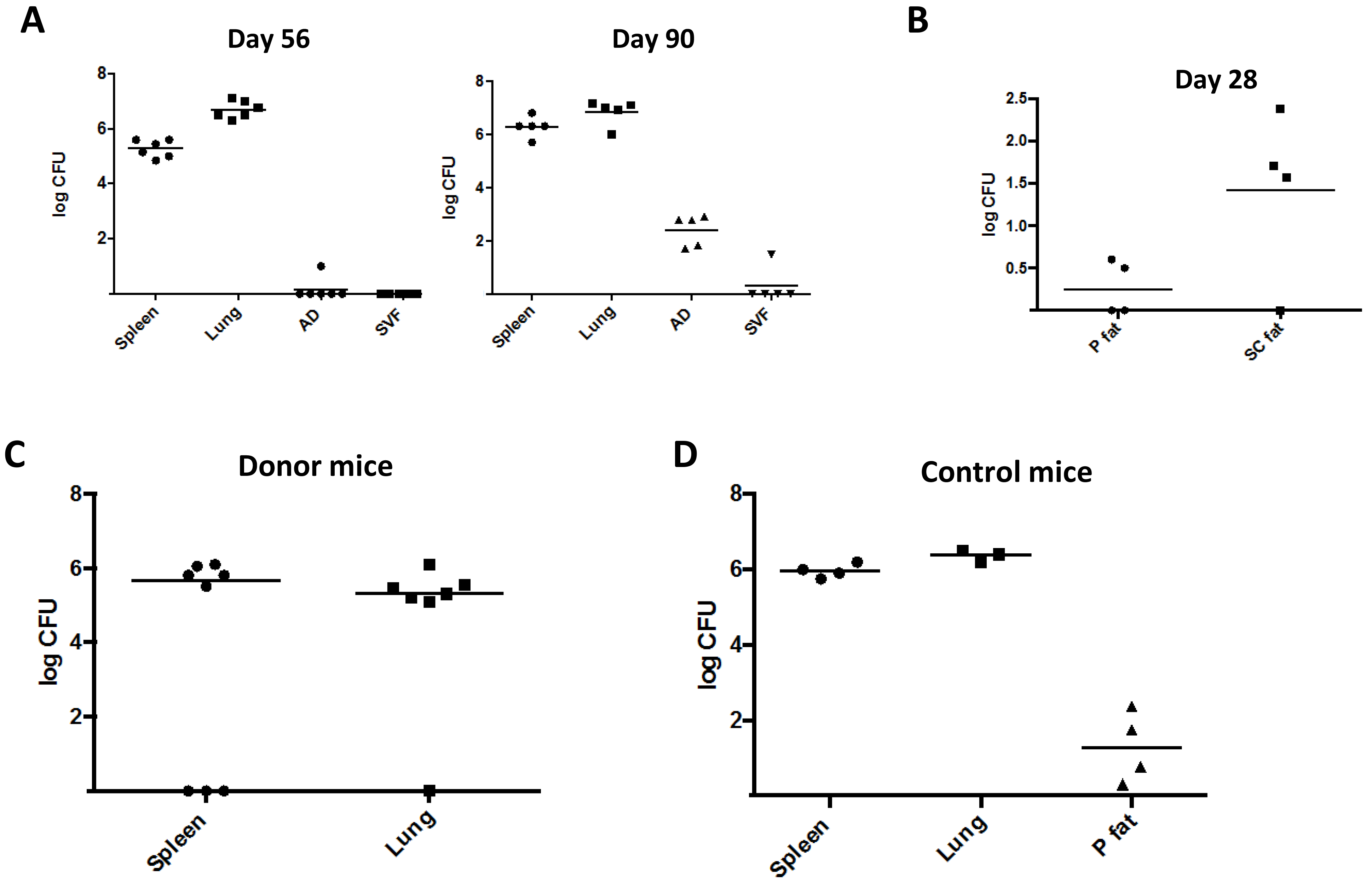

Supplement: S1 Fig — (A) log10 Mtb CFUs in in spleen, lung and AD and SVF fractions of perigonadal fat at different time points after aerosol infection. Data representative of four independent experiments (medians). (B) log10 Mtb CFUs in perigonadal and subcoutaneous fat at day 28 post aerosol-infection (200 CFUs). Data are representative of two independent experiments (medians). (C) log10 Mtb CFUs in spleen and lung from donor mice used for transfer experiments. Mice were infected i.v. with 5x10^6^ CFUs of Mtb, organs were collected 14 days after infection and perigonadal fat was transferred to uninfected recipient mice. Data representative of two independent experiments (medians). (D) log10 Mtb CFUs in spleen, lung, and perigonadal (P) fat from control mice infected i.v with 5x10^6^ CFUs of Mtb at the same time as the mice used for transfer experiments. Organs were collected 14 days after infection. Data representative of two independent experiments (medians). Abbreviations: AD, adipose fraction; P, perigonadal; SC, subcutaneous; SVF, stromal vascular fraction. (TIF) [file ppat.1006676.s001.tif]

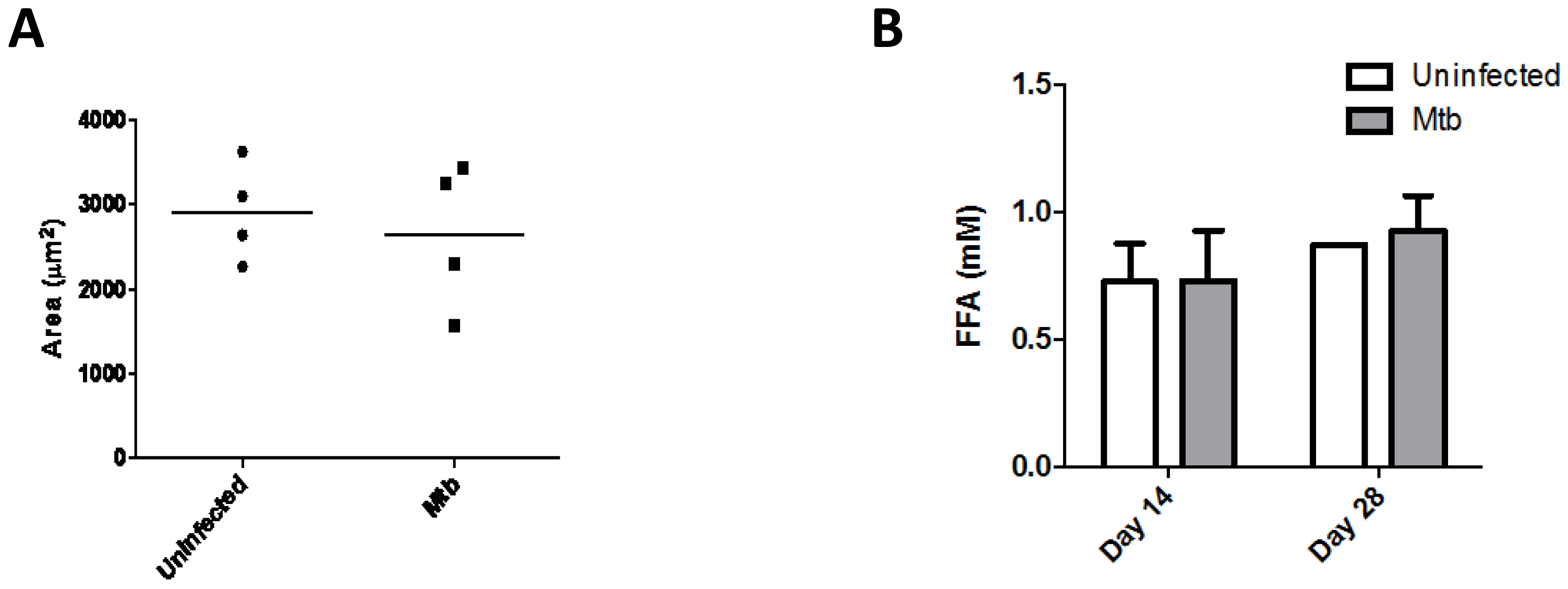

Supplement: S2 Fig — (A) Adipocyte size at day 28 post aerosol-infection. (B) Free fatty acids in sera at day 14 and 28 post infection. Data representative of two independent experiments. Abbreviations: FFA, free fatty acids. (TIF) [file ppat.1006676.s002.tif]

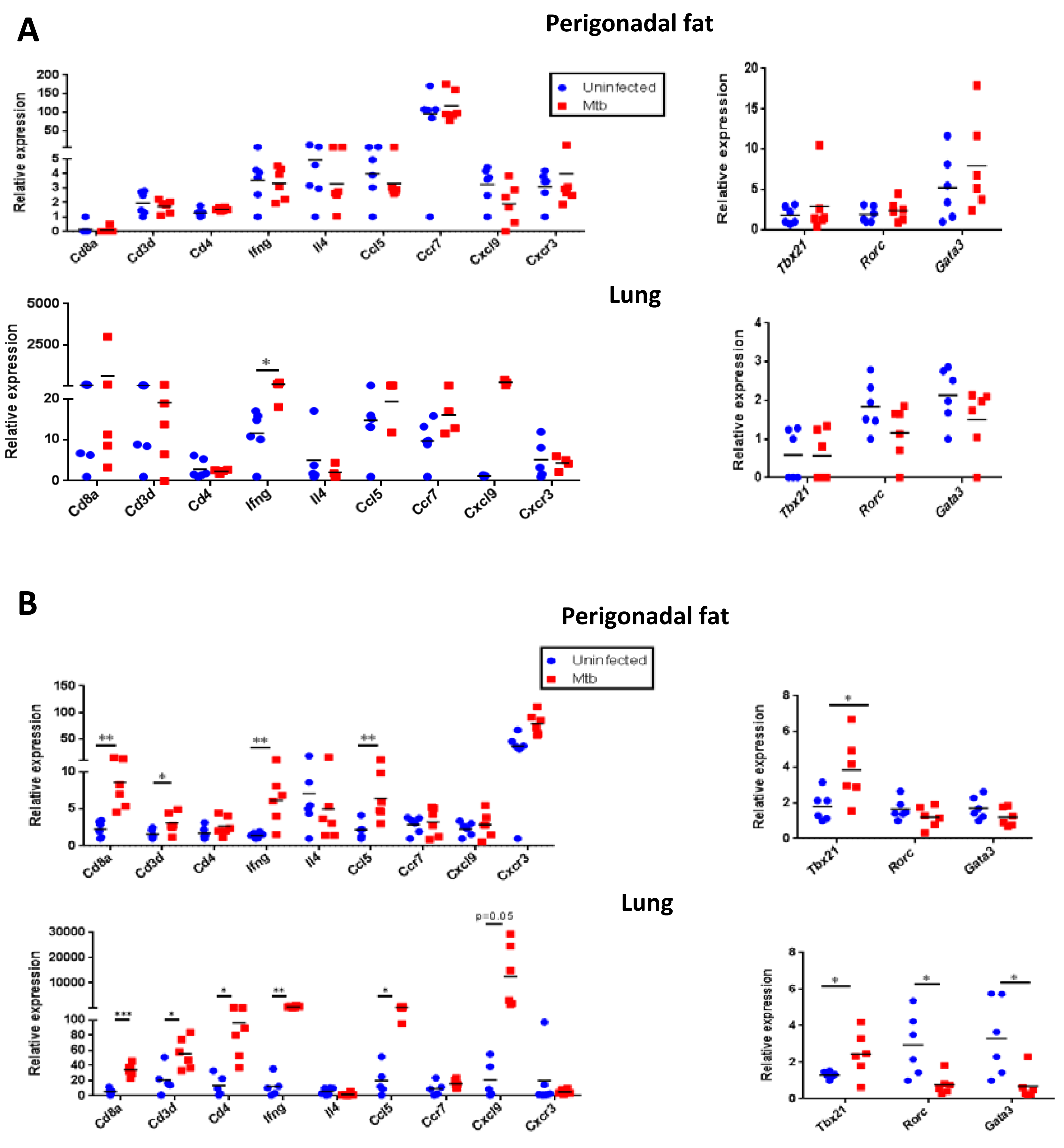

Supplement: S3 Fig — (A-B) Expression of Cd8a, Cd3d, Cd4, Ifng, Il4, Ccl5, Ccr7, Cxcl9, Cxcr3 (left panel) and Tbx21, Rorc and Gata3 (right panel) in perigonadal fat and lung, as measured with quantitative PCR at: (A) day 14 or (B) day 56 post infection. Data are representative of two to three independent experiments (means); *p<0.05, **p<0.01 and ***p<0.001 (Student’s t-test). (TIF) [file ppat.1006676.s003.tif]

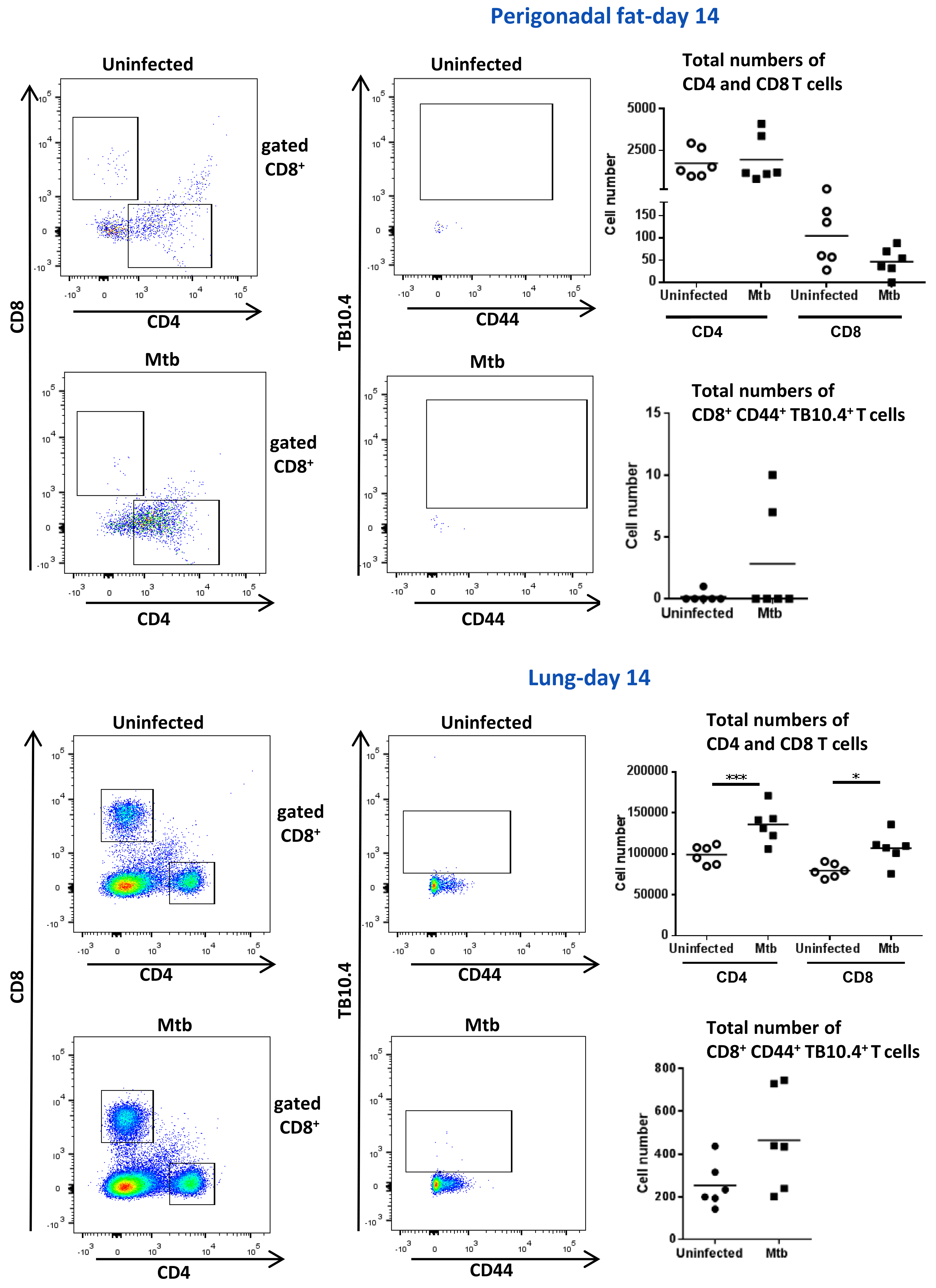

Supplement: S4 Fig — Numbers of CD4+, CD8+ and CD8+ CD44+ TB10.4+ (Mtb-specific) populations in SVF of perigonadal fat (upper panel) and lung (lower panel) at day 14 and post infection. Data are representative of two independent experiments (means); *p<0.05 and ***p<0.001 (Student´s t-test). Abbreviations: SVF, stromal vascular fraction. (TIF) [file ppat.1006676.s004.tif]

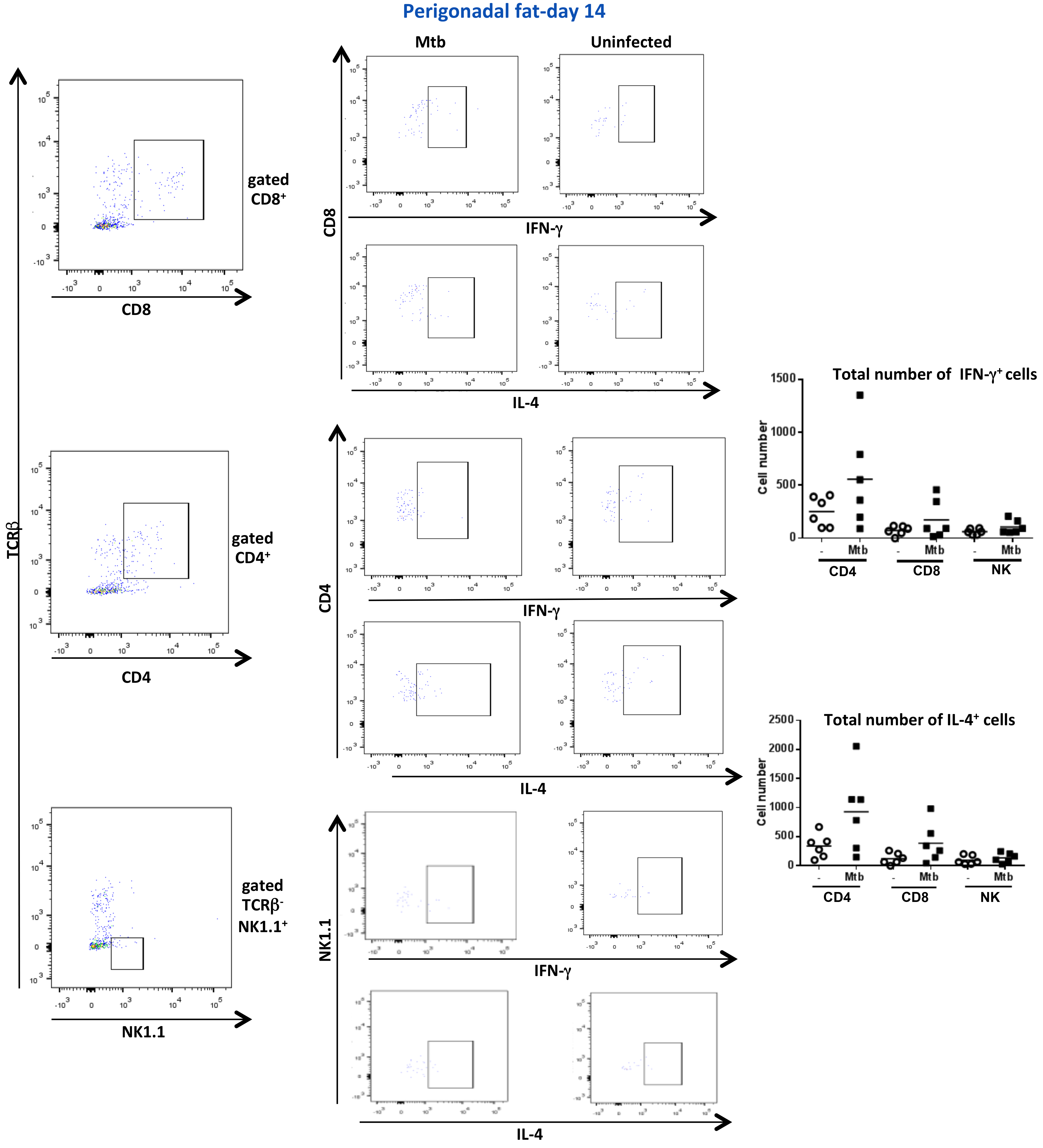

Supplement: S5 Fig — Numbers of CD4+, CD8+ and NK IFN-γ or IL-4-producing cells at day 14 post infection. Data are representative of two independent experiments (means). Abbreviations: SVF, stromal vascular fraction. (TIF) [file ppat.1006676.s005.tif]

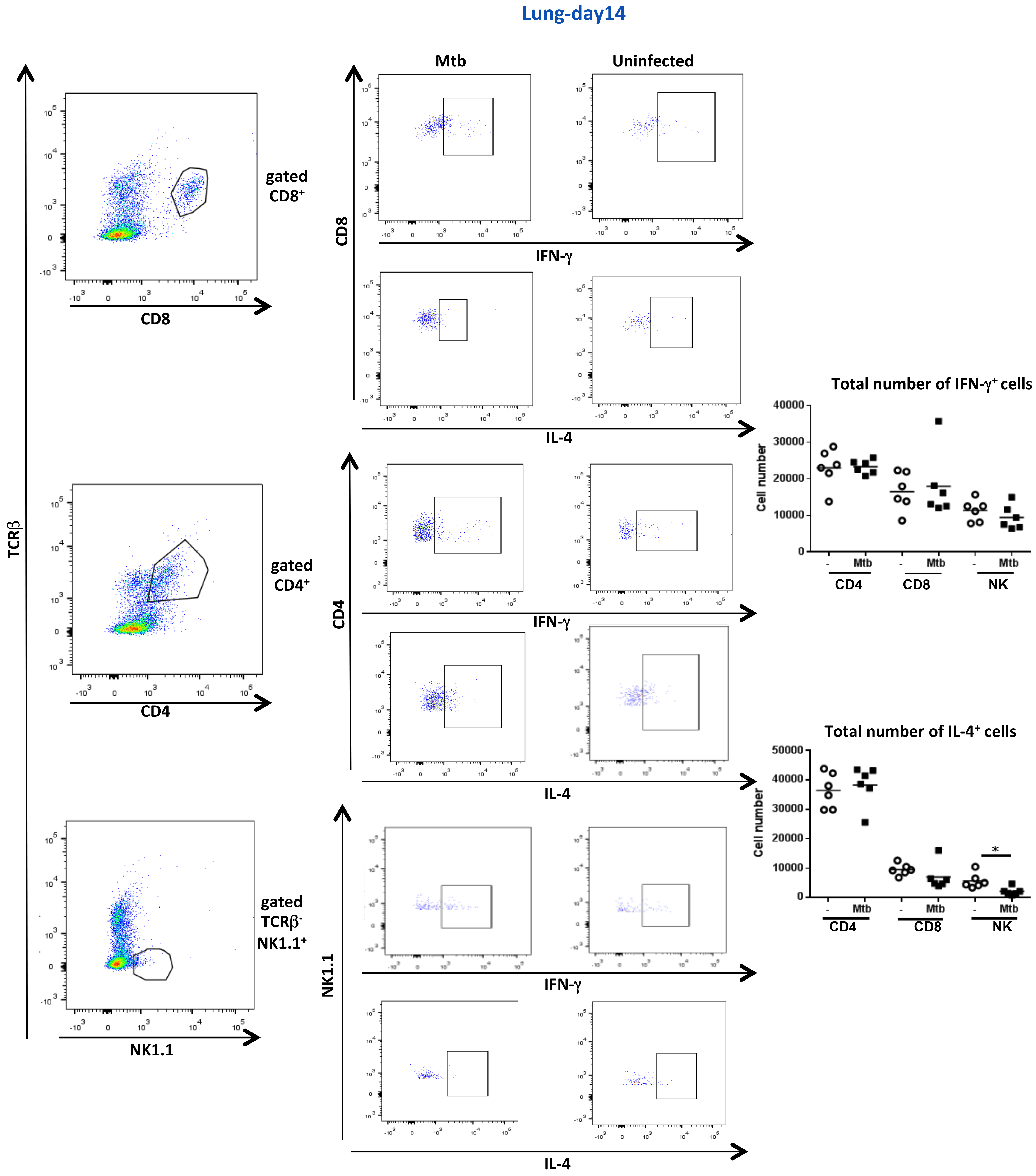

Supplement: S6 Fig — Numbers of CD4+, CD8+ and NK IFN-γ or IL-4-producing cells at day 14 post infection. Data are representative of two independent experiments (means); *p<0.05 (Student´s t-test). (TIF) [file ppat.1006676.s006.tif]

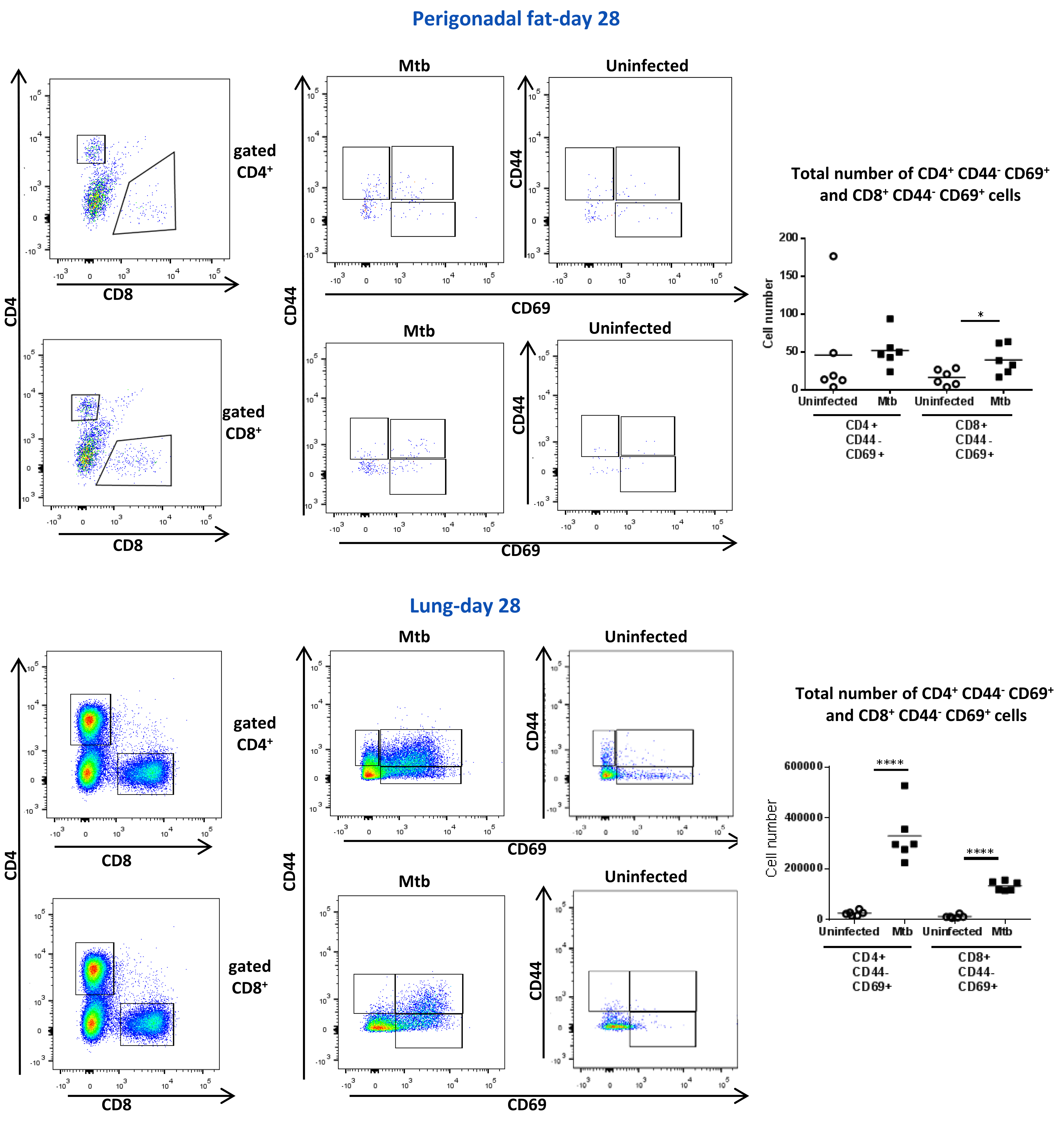

Supplement: S7 Fig — Numbers of CD4+ CD44– CD69+ and CD8+ CD44– CD69+ cells in SVF of perigonadal fat (upper panel) or lung (lower panel) at day 28 post infection. Data are representative of two independent experiments (means); *p<0.05, and ****p<0.0001 (Student´s t-test). Abbreviations: SVF, stromal vascular fraction. (TIF) [file ppat.1006676.s007.tif]

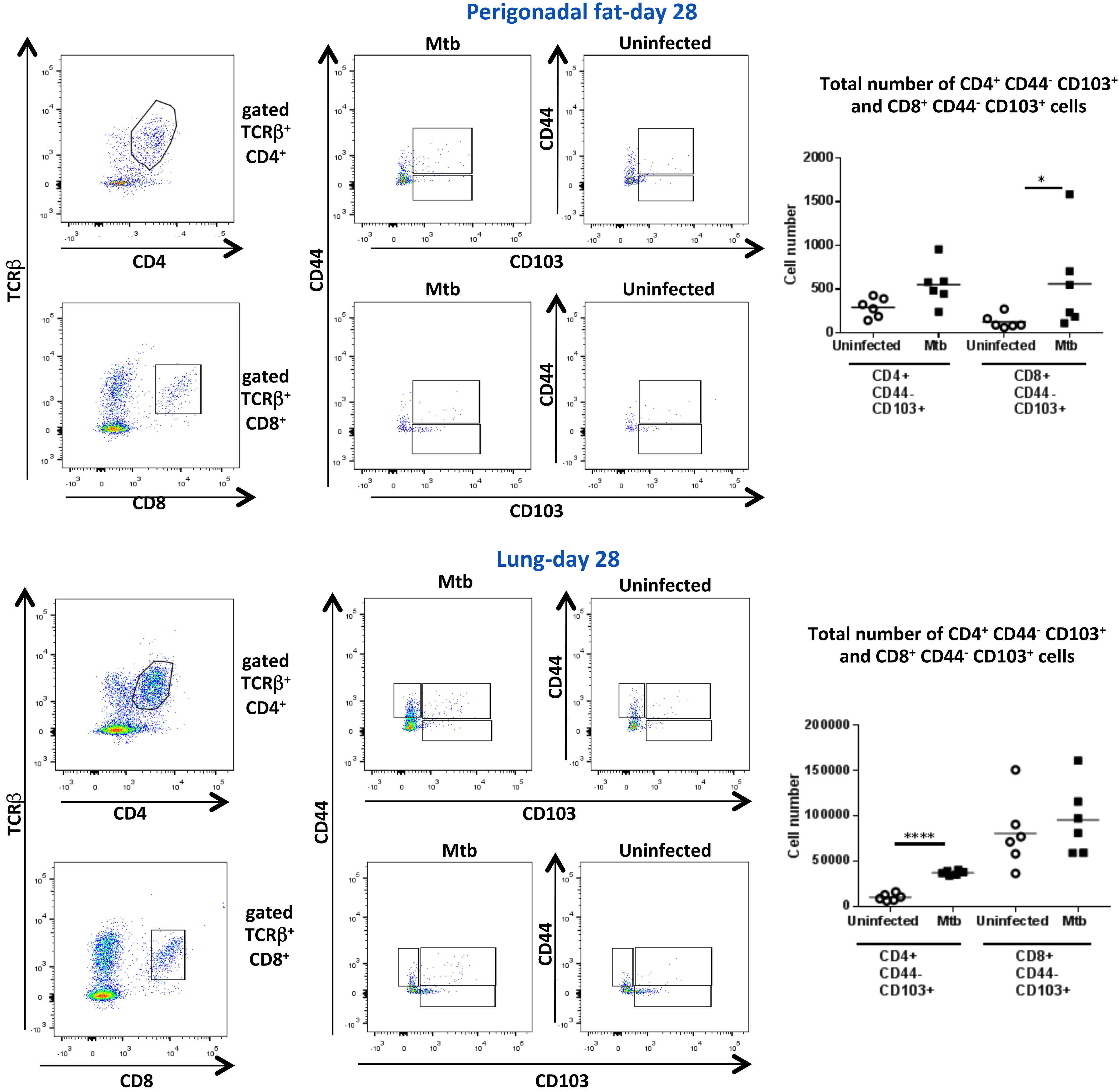

Supplement: S8 Fig — Numbers of CD4+ CD44– CD103+ and CD8+ CD44– CD103+ cells in SVF of perigonadal fat (upper panel) or lung (lower panel) at day 28 post infection. Data are representative of two independent experiments (means); *p<0.05, and ****p<0.0001 (Student´s t-test). Abbreviations: SVF, stromal vascular fraction. (TIF) [file ppat.1006676.s008.tif]

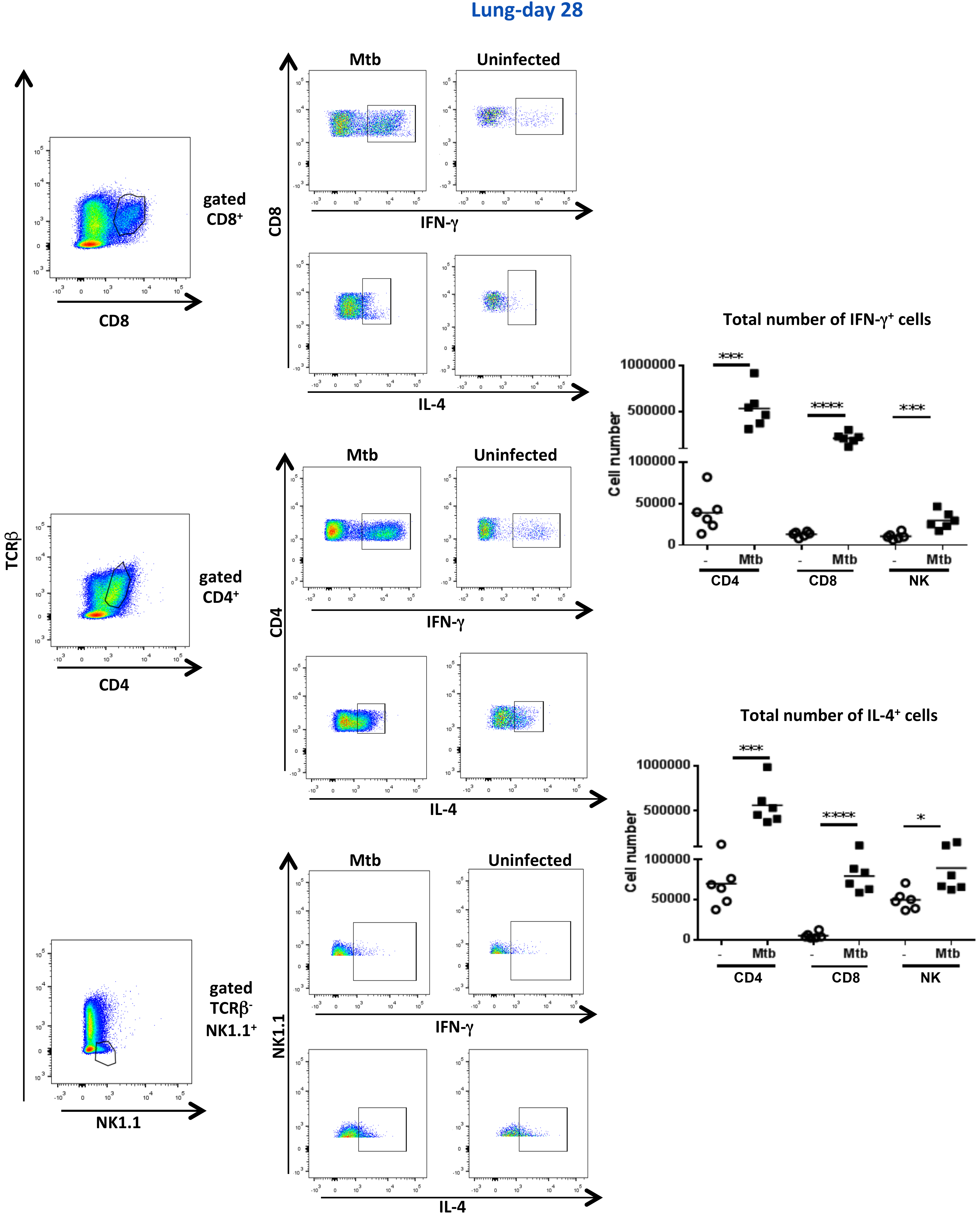

Supplement: S9 Fig — Numbers of CD4+, CD8+ and NK IFN-γ or IL-4-producing cells in lung at day 28 post infection. Data are representative of two independent experiments (means); *p<0.05, ***p<0.001 and ****p<0.0001 (Student´s t-test). (TIF) [file ppat.1006676.s009.tif]

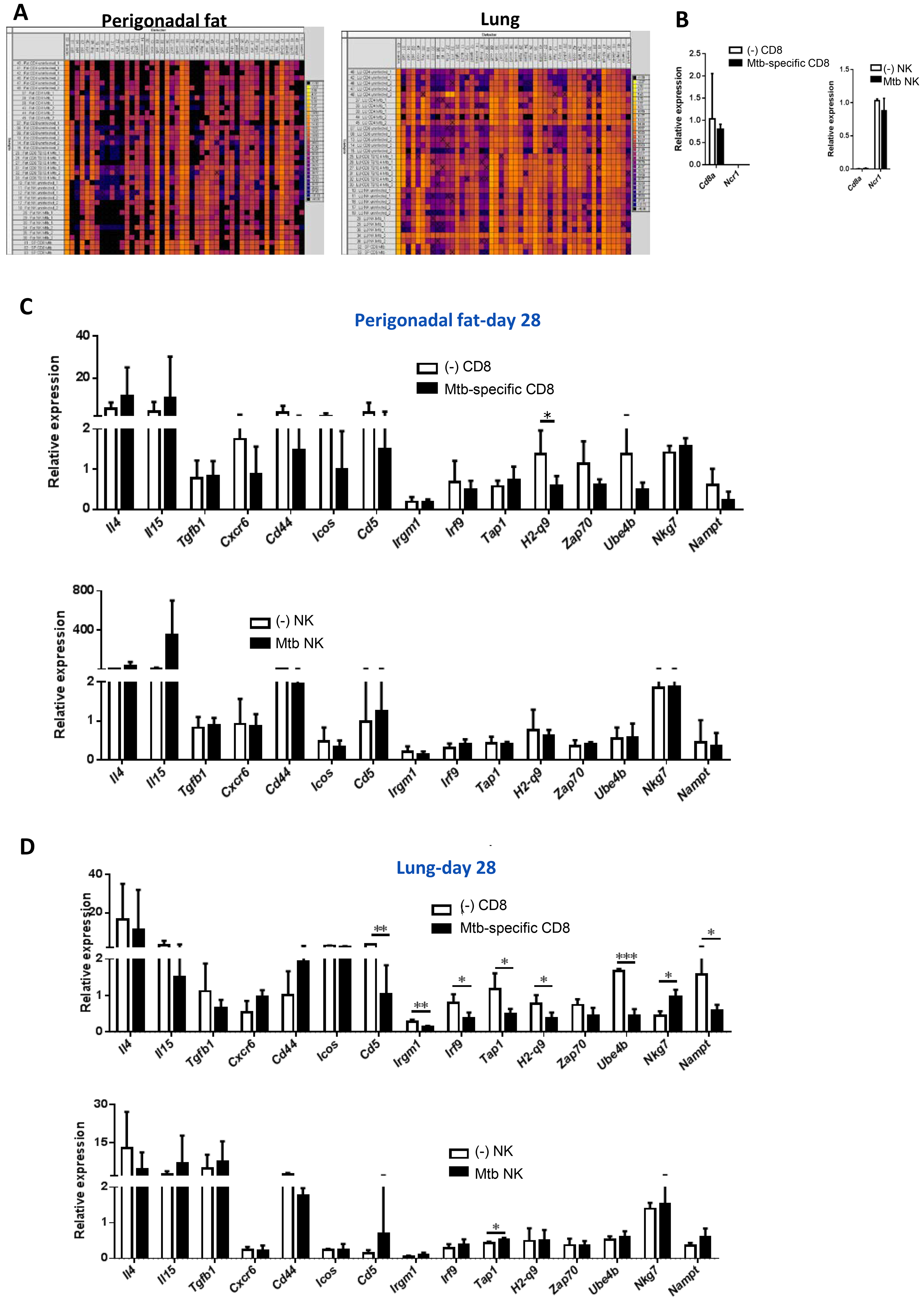

Supplement: S10 Fig — (A) Heat maps of gene expression of CD4+, CD8+, Mtb-specific CD8+ T cells (CD8+ CD44+ TB10.4+) and NK cells sorted from perigonadal fat (right panel) or lung (left panel) at day 28 post infection. Colour corresponds to ΔCt values. Mtb infection modulates gene expression in NK cells and Mtb-specific CD8+ T cells in perigonadal fat. (B-D) Relative gene expression of CD8+, CD8+ TB10.4 (Mtb-specific) and NK cells sorted from perigonadal fat (B-C) or lung (D) at day 28 post infection. Results of two independent experiments pooled (means); *p<0.05, **p<0.01 and ***p<0.001 (Student’s t-test). Abbreviations: (-), uninfected. (TIF) [file ppat.1006676.s010.tif]
